# Comparative efficacy of different interventions for post-stroke cricopharyngeal achalasia: a systematic review and network meta-analysis

**DOI:** 10.3389/fneur.2026.1802305

**Published:** 2026-04-10

**Authors:** Jianghuan Yue, Xiuli Wang, Cuiting Li, Lulu Wang, Xiaoling Li

**Affiliations:** Department of Rehabilitation Medicine, Lanzhou University Second Hospital, Lanzhou, Gansu, China

**Keywords:** balloon dilation, cricopharyngeal achalasia, dysphagia, network meta-analysis, stroke

## Abstract

**Objective:**

This study aims to employ network meta-analysis to systematically compare and evaluate the efficacy of various interventions for post-stroke cricopharyngeal achalasia.

**Methods:**

We searched 10 databases and trial registries from inception to December 3, 2025, to identify randomized controlled trials (RCTs) on cricopharyngeal achalasia after stroke. Two investigators independently screened studies, extracted data, and assessed risk of bias. The outcome indicators included effective rate, videofluoroscopic swallowing study (VFSS) score, Functional Oral Intake Scale (FOIS) score, and Standardized Swallowing Assessment (SSA) score.

**Results:**

A total of 36 articles covering 13 interventions were included. Network meta-analysis revealed: ① Regarding the evaluation of effective rate, the top three interventions based on SUCRA values were: balloon dilation combined with repetitive transcranial magnetic stimulation (rTMS) (91.8%) > balloon dilation combined with electromyographic biofeedback (EMGBF) (82.8%) > balloon dilation combined with acupuncture (79.4%); ② For the VFSS assessment, the highest SUCRA rankings were: balloon dilation combined with acupuncture (92.0%) > balloon dilation combined with tongue pressure resistance feedback (TPRF) (79.6%) > balloon dilation combined with botulinum toxin type A (BTX-A) (78.4%); ③ Concerning the FOIS evaluation, the top three SUCRA values were: balloon dilation combined with BTX-A (87.4%) > balloon dilation combined with transcranial direct current stimulation (tDCS) (83.0%) > balloon dilation combined with acupuncture (76.8%); ④ In the SSA evaluation, the leading interventions by SUCRA were: balloon dilation combined with acupuncture (80.0%) > balloon dilation combined with BTX-A (69.5%) > balloon dilation combined with rTMS (68.5%).

**Conclusion:**

Intervention efficacy varied across outcome measures. Balloon dilation combined with acupuncture may be the optimal intervention for improving VFSS scores and reducing SSA scores. Balloon dilation combined with BTX-A may be optimal for improving FOIS scores, and balloon dilation combined with rTMS may be optimal for increasing the effective rate. However, given the small sample sizes of included studies, inadequate allocation concealment and blinding, and potential publication bias for some outcomes, the present findings should be interpreted with caution. Further high-quality studies are warranted to validate these results.

**Systematic review registration:**

This systematic review has been prospectively registered in PROSPERO. Identifier CRD420251186725.

## 1 Introduction

Swallowing impairment represents a prevalent complication following stroke, exhibiting a comparatively high incidence rate among stroke survivors (1). Cricopharyngeal achalasia (CPA), as a significant manifestation of post-stroke dysphagia, demonstrates an incidence rate ranging from 6 to 61% (2). CPA is characterized by the failure of the cricopharyngeal muscle to relax in a timely manner or by abnormally elevated muscle tone during swallowing, manifesting as functional impairments including complete failure to open, incomplete opening, or uncoordinated opening (3). CPA may result in dysphagia, aspiration pneumonia, dehydration, and malnutrition. Not only does it diminish patients’ quality of life, but it may also lead to severe complications such as asphyxia (4). Consequently, investigating safe and effective therapeutic interventions is of considerable clinical importance.

Regarding the treatment of CPA, currently, common clinical treatment regimens include rehabilitation training, neuromuscular electrical stimulation, balloon dilation, cricopharyngeal myotomy, among others (5, 6), as well as the combined application of various treatment methods. All these interventions can promote the recovery of swallowing function. However, there is currently no consensus on the optimal treatment regimen for CPA. Therefore, this study used the network meta-analysis method, combining direct and indirect comparisons, to systematically evaluate the intervention effects of different interventions on CPA after stroke. Furthermore, therapeutic outcomes were ranked across four parameters: effective rate, videofluoroscopic swallowing study (VFSS) score, Functional Oral Intake Scale (FOIS) score, and Standardized Swallowing Assessment (SSA) score.

## 2 Methods

### 2.1 Study design and registration

The current investigation followed the methodological standards outlined in the PRISMA framework (7) and the Cochrane Handbook to perform a comprehensive systematic analysis, maintaining strict research protocols. Furthermore, this research project was officially registered in the PROSPERO database (Registration ID: CRD420251186725) on November 8, 2025. Publicly Accessible URL: https://www.crd.york.ac.uk/PROSPERO/view/CRD420251186725.

### 2.2 Literature search

Literature retrieval was independently conducted by two investigators in strict accordance with the predefined research protocol. The search period spanned from the inception of each respective database to December 3, 2025. Chinese databases searched included the China National Knowledge Infrastructure (CNKI), Wanfang Data, VIP Database for Chinese Technical Periodicals (VIP), and the Chinese Biomedical Literature Database (CBM); English databases included PubMed, Web of Science, Embase, the Cochrane Library, Scopus, and ProQuest. The search terms were “stroke” and “cricopharyngeal achalasia,” with study type restricted to randomized controlled trials (RCTs). The detailed search strategy is provided in Supplementary Tables 1–10. Additionally, a manual search of clinical trial registries was undertaken, along with a supplementary manual review of reference lists from relevant studies.

### 2.3 Inclusion criteria

(1) Research Type: RCTs, with language restricted to Chinese and English.
(2) Research Subjects: Subjects must meet the clear diagnostic criteria for stroke, be diagnosed with cerebral hemorrhage or cerebral infarction through imaging examinations such as head CT or magnetic resonance imaging (MRI), and have cricopharyngeal achalasia confirmed by VFSS. No restrictions were applied with respect to gender or disease duration; however, participants were required to be at least 18 years of age.
(3) Intervention Measures: The control group received conventional dysphagia training (CDT), primarily comprising foundational swallowing exercises and feeding therapy. The intervention group combined different types of stimulation therapies on the basis of the control group, including balloon dilatation, acupuncture, neuromuscular electrical stimulation (NMES), repetitive transcranial magnetic stimulation (rTMS), transcranial direct current stimulation (tDCS), tongue pressure resistance feedback (TPRF), botulinum toxin type A (BTX-A), electromyographic biofeedback (EMGBF), and their combined regimens. Direct comparative studies between the aforementioned different interventions were also eligible.
(4) Outcome Measures: effective rate, videofluoroscopic swallowing study (VFSS), Functional Oral Intake Scale (FOIS) score, and Standardized Swallowing Assessment (SSA) score.

The effective rate in this study was defined as follows: it was calculated based on the therapeutic effect evaluation criteria of VFSS (see Supplementary Table 11), using the formula: Effective rate = (Number of cured cases + Number of effective cases)/Total number of cases × 100%. Alternatively, it was determined according to the grading criteria of the Kubota water swallowing test (see Supplementary Table 12), using the formula: Effective rate = (Number of cured cases + Number of markedly effective cases + Number of effective cases)/Total number of cases × 100%. Original studies reporting effective rate outcomes are presented in Supplementary Table 13.

### 2.4 Exclusion criteria

(1) Animal experimentation.
(2) Conference proceedings or dissertations.
(3) Incomplete raw data or inability to extract valid data.
(4) Non-Chinese and non-English literature.

### 2.5 Data extraction

Two researchers separately evaluated the collected studies and gathered pertinent information, subsequently engaging in a cross-verification procedure. Should discrepancies arise, a third researcher was consulted to achieve consensus. The specific protocol was as follows: Firstly, transfer identified publications into EndNote X9 to eliminate duplicate entries. Secondly, perform initial screening through title and abstract review. Lastly, conduct full-text assessment for secondary screening. The extracted information primarily included bibliographic details, intervention specifications, outcome measures, and adverse events.

### 2.6 Quality assessment

The quality of the included studies was assessed using the Cochrane Risk of Bias Assessment Tool (8). The methodological quality and risk of bias were assessed across seven domains, including random sequence generation, allocation concealment, blinding, outcome assessment, data integrity, follow-up integrity, and other biases. Judgments were presented as “low risk, high risk, or unclear.” Additionally, the revised Jadad Scale was employed to evaluate the literature quality from four aspects: the method of random sequence generation, allocation concealment, blinding, and withdrawals and drop-outs. The first three items were scored from 0 to 2 points each, and the last item was scored from 0 to 1 point. A total score of 1–3 points indicated low-quality studies, while a score of 4–7 points indicated high-quality studies (9).

### 2.7 Statistical analysis

In the assessment of study results, various statistical measures were employed according to variable types. Dichotomous outcomes were analyzed using odds ratios (OR) with their respective 95% confidence intervals (CIs). For continuous variables, including VFSS, FOIS, and SSA, mean differences (MD) with 95% CIs served as the primary effect measures. The means and standard deviations (SD) for continuous outcome variables before and after treatment were computed according to the guidelines outlined in Section 16.1.3.2 of the Cochrane Handbook version 5.0.2. The research team utilized RevMan 5.3 software for both risk of bias assessment in literature screening and conventional meta-analyses. Evaluation of data heterogeneity involved examination of I^2^ statistics, with a fixed-effect analytical approach being implemented when the I^2^ value remained below 50% and the associated *p*-value exceeded 0.05. When the heterogeneity index (I^2^) exceeded 50% with a *p*-value below 0.05, substantial variation was observed across the selected research studies. To investigate the origins of this variability, sensitivity assessments and subgroup examinations were performed. In cases where heterogeneity persisted despite these measures, a random-effects approach was employed for comprehensive evaluation (10). This study employed a network meta-analysis model within the framework of the frequentist school. Its core premise was the transitivity assumption, which posits that among different intervention comparison groups, the distributions of clinical and methodological characteristics influencing the outcome effects are balanced, enabling reliable indirect inferences through a common control intervention. To validate the transitivity assumption, we first conducted a comparability analysis of important covariates such as the key baseline characteristics (e.g., mean age, gender ratio, stroke duration) of the included studies and the intervention duration. Box plots were used to display the distribution balance of each covariate to ensure good clinical comparability among different intervention comparisons. Subsequently, Stata 14.0 was used to perform inconsistency assessment, including global and local inconsistency tests. If the *p*-value was greater than 0.05, no statistically significant inconsistency between direct and indirect comparisons was found. Furthermore, the consistency of the closed-loop of each outcome indicator was evaluated through the loop inconsistency test. If the 95% confidence interval of the loop inconsistency factor contained 0, it indicated that the direct and indirect evidence were consistent. On the premise that no obvious inconsistency was found, this study used a consistency model for analysis. The relatively optimal intervention measures were determined through result tables and cumulative probability ranking plots, and publication bias was evaluated using funnel plots and Egger’s test, with the significance level set at *α* = 0.05.

## 3 Results

### 3.1 Search results

**Figure 1:**
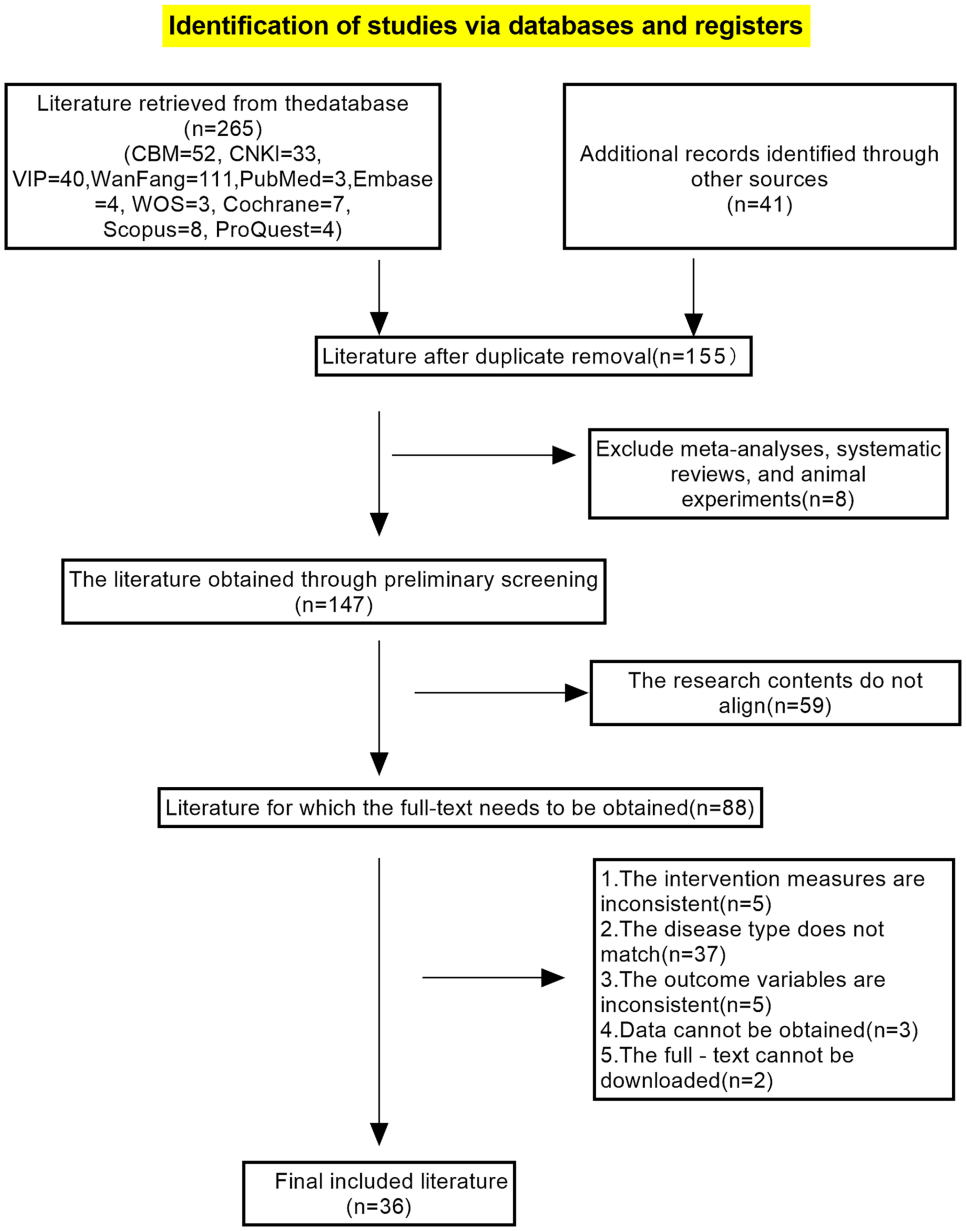
Flowchart for literature screening.

A preliminary search yielded 316 studies, and after reading and screening, a total of 36 articles were included. The specific screening process is shown in Figure 1.

### 3.2 Characteristics of the included studies

This review included 36 studies published between 2015 and 2025, involving 1,816 stroke patients diagnosed with cricopharyngeal achalasia. The study population comprised 944 participants in the intervention group and 872 in the control group. All selected publications were RCTs, with their baseline characteristics summarized in Table 1 (11–46).

**Table 1:** Characteristics of included studies.

Author and year	Sample size (m/f)		Mean age (year)		Disease duration		Treatment course	Intervening measure		Outcome
	T	C	T	C	T	C		T	C	
Zhang (2019) (11)	18 (10/8)	18 (9/9)	61 ± 9	59.3 ± 10.1	23.9 ± 16.1 (d)	25.3 ± 18.7 (d)	20d	BD + Acu	BD	①②
Wang and Liu (2023) (12)	40 (16/24)	40 (22/18)	48.1 ± 3.0	46.8 ± 2.4	29.5 ± 3.1 (d)	33.2 ± 2.7 (d)	4w	BD + Acu	BD	①②④
Liu et al. (2023) (13)	14 (11/3)	15 (10/5)	65.8 ± 5.7	67.6 ± 6.1	87.2 ± 15.7 (d)	90.7 ± 14.2 (d)	2w	BD + (BTX-A)	BD	①③
He et al. (2016) (14)	16 (11/5)	16 (12/4)	65.36 ± 8.67	66.75 ± 7.13	2.61 ± 2.74 (m)	2.32 ± 2.88 (m)	2w	BD	CDT	②
Zhang et al. (2025) (15)	40 (22/18)	40 (24/16)	66.59 ± 4.55	66.38 ± 4.40	3.35 ± 0.31 (m)	3.29 ± 0.29 (m)	2w	BD + NMES	CDT	②③
Lin and Zheng (2018) (16)	25 (14/11)	25 (15/10)	52.6 ± 5.5	51.4 ± 4.9			6w	BD	CDT	①②③
Wang et al. (2023) (17)	24 (16/8)	24 (18/6)	61.7 ± 2.6	60.5 ± 2.8	33.5 ± 2.7 (d)	33.8 ± 2.4 (d)	4w	BD + NMES	CDT	①③
Huang et al. (2017) (18)	20 (12/8)	20 (13/7)	60.5 ± 3.4	60.8 ± 3.1	35.6 ± 10.9 (d)	35.2 ± 10.7 (d)	3w	BD + NMES	NMES	①②
Huang et al. (2016) (19)	19 (12/7)	19 (10/9)	61 ± 11	63 ± 15	4.2 ± 2.7 (m)	3.8 ± 2.4 (m)	4w	BD	CDT	①②③
Long et al. (2021) (20)	30 (19/11)	30 (18/12)	60.53 ± 10.61	59.93 ± 12.89	21.73 ± 18.07 (d)	22.07 ± 16.74 (d)	4w	BD + Acu	BD	④
Cao et al. (2019) (21)	30 (17/13)	30 (16/14)	61 ± 5	61 ± 5	30.86 ± 0.52 (d)	30.57 ± 0.64 (d)	4w	BD + Acu	BD	④
Yang and Chen (2017) (22)	24 (14/10)	24 (13/11)	60 ± 8	60 ± 5	4.57 ± 0.64 (d)	4.86 ± 0.52 (d)	4w	BD + Acu	BD	④
Mai (2019) (23)	40 (22/18)	40 (21/19)	63.8 ± 9.4	64.0 ± 9.1	78.75 ± 12.64 (d)	78.56 ± 13.59 (d)	4w	BD	CDT	②
Luo et al. (2020) (24)	34 (18/16)	33 (19/14)	63.3 ± 7.3	62.6 ± 8.1	28.2 ± 6.8 (d)	29.4 ± 5.8 (d)	4w	BD + Acu	BD	①②
Gao (2020) (25)	24 (16/8)	24 (15/9)	52.68 ± 4.75	50.85 ± 4.69	4.82 ± 0.24 (w)	4.45 ± 0.16 (w)	4w	BD + Acu	BD	①②④
Zhao et al. (2020) (26)	5 (4/1)	5 (3/2)	53.48 ± 3.25	53.54 ± 3.36	108.00 ± 3.71 (d)	112.00 ± 3.18 (d)	4w	BD + (BTX-A)	BD	②④
Wang et al. (2020) (27)	T1:10 (7/3)	10 (6/4)	59.43 ± 10.35	58.00 ± 9.59	33.00 ± 12.26 (d)	32.00 ± 11.71 (d)	8w	TPRF	BD	②③
	T2:10 (6/4)		58.67 ± 10.10		33.00 ± 10.32 (d)			BD + TPRF		
Zhang et al. (2018) (28)	16 (10/6)	16 (14/2)	63.56 ± 11.09	65.06 ± 8.03	2.72 ± 2.88 (d)	3.01 ± 2.44 (d)	6w	BD + NMES	NMES	②
Wang et al. (2024) (29)	T1:15 (9/6)	15 (9/6)	54.46 ± 9.02	57.73 ± 6.99	49.00 ± 9.66 (d)	55.93 ± 9.20 (d)	3w	Acu	BD	②③
	T2:15 (10/5)		52.06 ± 9.12		54.93 ± 12.38 (d)			BD + Acu		
Tang et al. (2017) (30)	44 (29/15)	44 (28/16)	51.3 ± 10.4	51.1 ± 10.5			6w	BD	CDT	①②
Li et al. (2019) (31)	T1:15 (8/7)	15 (7/8)	57 ± 8	57 ± 7	2.5 ± 1.4 (m)	2.2 ± 1.1 (m)	6w	BD	CDT	②
	T2:15 (8/7)		56 ± 8		2.5 ± 1.6 (m)			BD + Acu		
Wang et al. (2021) (32)	30 (16/14)	30 (14/16)	59.71 ± 10.58	58.42 ± 12.54	3.18 ± 1.67 (d)	3.25 ± 1.64 (d)	4w	rTMS	BD	①④
	30 (13/17)		60.28 ± 11.49		3.63 ± 1.54 (d)			BD + rTMS		
Zhang (2017) (33)	20 (14/6)	20 (12/8)	63.56 ± 8.03	65.06 ± 11.09	2.72 ± 2.44 (m)	3.01 ± 2.88 (m)		BD + Acu	Acu	②
Zhuang et al. (2015) (34)	30	30	62.0 ± 3.2	62.0 ± 3.2				BD	CDT	①
Zhu et al. (2018) (35)	25 (15/10)	25 (14/11)	59.27 ± 11.55	58.96 ± 11.62	45.0 ± 23.8 (d)	40.0 ± 25.1 (d)	4w	BD + NMES	NMES	①②③
Fan et al. (2020) (36)	33 (18/15)	33 (18/15)	67.31 ± 5.36	66.58 ± 5.83	1.35 ± 0.59 (m)	1.29 ± 0.42 (m)	2 m	BD + Acu	BD	①②③
Zhang et al. (2015) (37)	25 (15/10)	25 (14/11)	62.41 ± 5.10	62.36 ± 5.02			15d	BD	CDT	①
Zhao et al. (2016) (38)	15	15						BD	NMES	①
Wang et al. (2023) (39)	25 (16/9)	24 (18/6)	59.48 ± 11.97	61.04 ± 9.43	41.92 ± 15.23 (d)	42.46 ± 14.02 (d)	3w	BD + rTMS	BD	②③
Shao et al. (2017) (40)	32 (21/11)	32 (22/10)	61.09 ± 6.82	62.78 ± 7.08	36.09 ± 10.81 (d)	37.44 ± 10.44 (d)	4w	BD	CDT	①
Wang et al. (2023) (41)	42 (22/20)	42 (24/18)	74.57 ± 2.21	74.32 ± 2.31			20d	BD	CDT	①
Wang et al. (2020) (42)	14 (11/3)	14 (10/4)	61.43 ± 11.237	62.00 ± 10.459	66.79 ± 38.623 (d)	67.50 ± 47.622 (d)	20d	BD + tDCS	BD	③
Xu (2025) (43)	24 (15/9)	24 (12/12)	70.13 ± 11.35	70.67 ± 11.43	13.96 ± 5.61 (d)	13.33 ± 2.53 (d)	4w	BD + Acu	BD	③④
Wei et al. (2016) (44)	10	10	48.85 ± 9.13	48.85 ± 9.13	3.40 ± 1.14 (m)	3.40 ± 1.14 (m)	6w	BD	CDT	②
Zhang et al. (2021) (45)	15 (10/5)	15 (9/6)	65.47 ± 5.33	65.23 ± 5.12	2.26 ± 0.15 (m)	2.11 ± 0.12 (m)		BD	CDT	①④
Jing et al. (2025) (46)	30 (16/14)	30 (18/12)	73.3 ± 3.5	73.2 ± 4.3	32.67 ± 8.05 (d)	32.49 ± 7.12 (d)	4w	BD + EMGBF	BD	①②④

T, treatment group; C, control group; m/f, male and female; m, month; d, day; w, week; TPRF, tongue pressure resistance feedback; NMES, neuromuscular electrical stimulation; tDCS, transcranial direct current stimulation; rTMS, repetitive transcranial magnetic stimulation; EMGBF, electromyographic biofeedback; BTX-A, botulinum toxin type A; CDT, conventional dysphagia training; BD, balloon dilation; Acu, acupuncture; ①: effective rate; ②: videofluoroscopic swallowing study (VFSS); ③: Functional Oral Intake Scale (FOIS); ④: Standardized Swallowing Assessment (SSA).

### 3.3 Risk of bias

**Figure 2:**
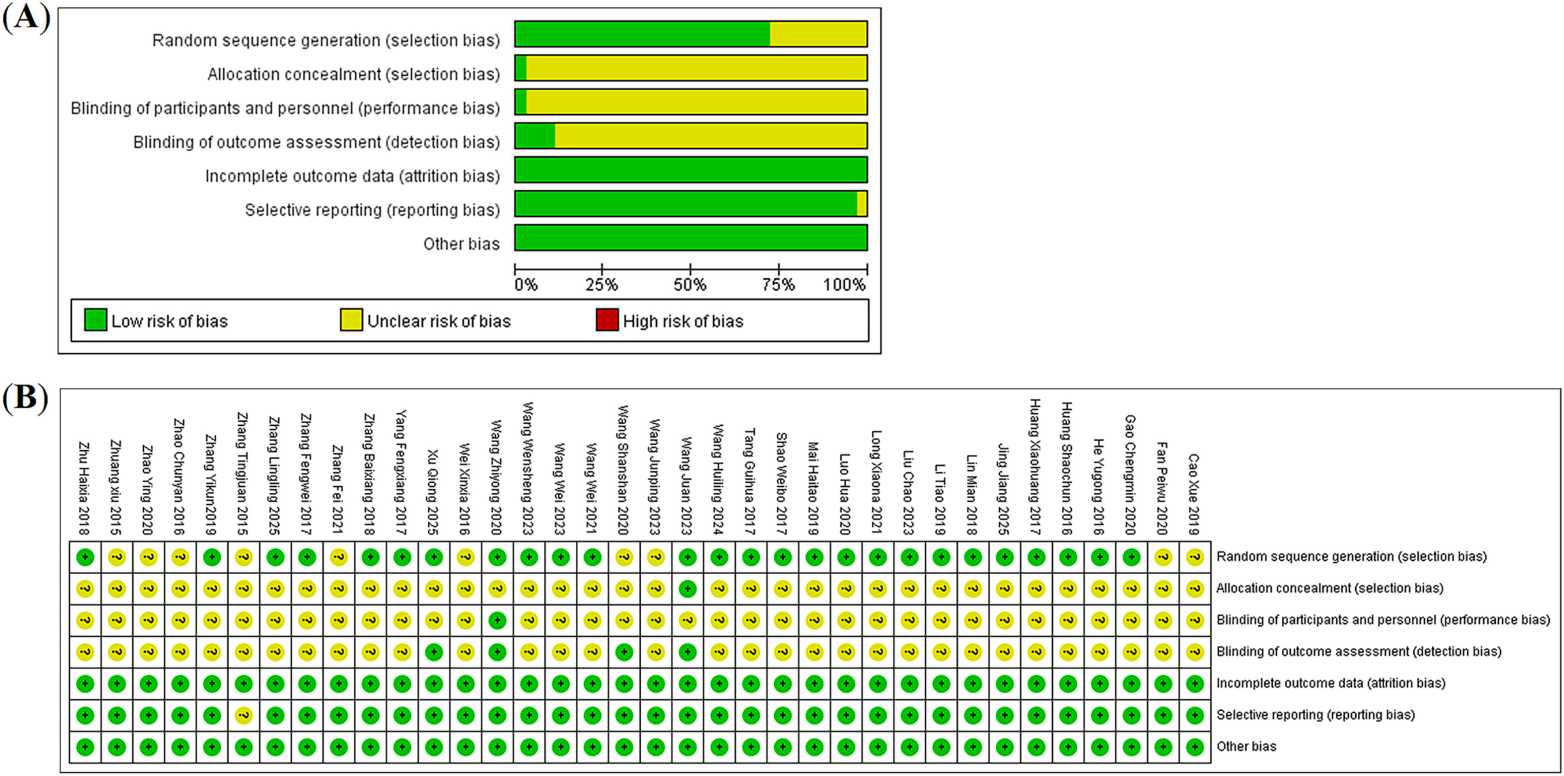
Risk of bias assessment results of the included studies: **(A)** risk of bias graph and **(B)** risk of bias summary.

Randomization was mentioned in all 36 included studies. Specifically, 10 of them did not explicitly report the specific randomization procedure (17, 21, 26, 27, 34, 36–38, 44, 45); one study employed the allocation concealment method (39); and four publications implemented the blinding design (27, 39, 42, 43), while the remaining were not specified. None of the studies showed selective reporting or missing outcome data, and no other sources of bias were mentioned in the texts. Figure 2 presents the comprehensive distribution of these findings. The modified Jadad scale was employed for quality evaluation, and the results indicated that there were 26 high-quality and 10 low-quality studies. For detailed information, please refer to Supplementary Table 14.

### 3.4 Pairwise meta-analysis

#### 3.4.1 Effective rate

For the outcome of effective rate, a total of 18 studies were included, involving 9 intervention protocols. A fixed-effects model was used due to low heterogeneity across studies (I^2^ < 50%, *p* ≥ 0.05).

The results indicated that regarding the improvement of effective rate: balloon dilation combined with acupuncture, balloon dilation combined with rTMS, and balloon dilation combined with EMGBF were all statistically superior to balloon dilation alone; balloon dilation combined with NMES and balloon dilation alone were superior to CDT; balloon dilation combined with rTMS was superior to rTMS alone; balloon dilation combined with NMES was superior to NMES alone. All the above differences were statistically significant (*p* < 0.05). A summary of the results can be found in Table 2, and the specific pairwise meta-analysis results are presented in Supplementary Figure 1.

**Table 2:** Pairwise meta-analysis.

Comparison	Number of studies	Heterogeneity test		Results of the meta-analysis	
		*P*-value	I^2^ (%)	OR/MD (95%CI)	*P*-value
Effective rate					
BD + Acu/BD	5	0.92	0	4.95 (2.30, 10.63)	<0.0001
BD + NMES/CDT	1	NR	NR	6.60 (1.25, 34.95)	0.03
BD + NMES/NMES	2	0.75	0	7.25 (1.89, 27.77)	0.004
BD + (BTX-A)/BD	1	NR	NR	2.00 (0.38, 10.51)	0.41
BD/CDT	4	0.96	0	8.30 (3.33, 20.69)	<0.00001
rTMS/BD	1	NR	NR	0.74 (0.25, 2.17)	0.58
BD + rTMS/BD	1	NR	NR	12.43 (1.46, 105.74)	0.02
BD + rTMS/rTMS	1	NR	NR	16.79 (2.00, 140.90)	0.009
BD/NMES	1	NR	NR	4.33 (0.71, 26.53)	0.11
BD + EMGBF/BD	1	NR	NR	6.00 (1.17, 30.72)	0.03
VFSS					
BD + Acu/BD	7	0.58	0	2.09 (1.75, 2.43)	<0.00001
BD + NMES/CDT	1	NR	NR	1.71 (1.29, 2.13)	<0.00001
BD + NMES/NMES	3	0.59	0	2.29 (1.83, 2.74)	<0.00001
BD + (BTX-A)/BD	1	NR	NR	1.92 (2.29, 6.13)	0.37
BD/CDT	7	0.77	0	2.54 (2.22, 2.86)	<0.00001
TPRF/BD	1	NR	NR	−2.90 (−4.08, −1.72)	<0.00001
BD + TPRF/BD	1	NR	NR	1.50 (0.21, 2.79)	0.02
BD + TPRF/TPRF	1	NR	NR	4.40 (3.31, 5.49)	<0.00001
Acu/BD	1	NR	NR	0.14 (−1.38, 1.66)	0.86
BD + Acu/Acu	2	0.91	0	1.44 (0.18, 2.71)	0.03
BD + Acu/CDT	1	NR	NR	4.47 (3.43, 5.51)	<0.00001
BD + rTMS/BD	1	NR	NR	1.01 (−0.93, 2.95)	0.31
BD + EMGBF/BD	1	NR	NR	0.65 (−0.53, 1.83)	0.28
FOIS					
BD + Acu/BD	3	1.00	0	1.16 (0.56, 1.76)	0.0002
BD + NMES/CDT	3	0.05	67	1.08 (0.11, 2.05)	0.03
BD + NMES/NMES	1	NR	NR	1.32 (0.43, 2.21)	0.004
BD + (BTX-A)/BD	1	NR	NR	1.73 (0.53, 2.93)	0.005
BD/CDT	1	NR	NR	2.62 (2.24, 3.00)	<0.00001
TPRF/BD	1	NR	NR	−1.60 (−2.60,-0.60)	0.002
BD + TPRF/BD	1	NR	NR	1.10 (0.01, 2.19)	0.05
BD + TPRF/TPRF	1	NR	NR	2.70 (1.73, 3.67)	<0.00001
Acu/BD	1	NR	NR	0.14 (−1.11, 1.39)	0.83
BD + Acu/Acu	1	NR	NR	1.00 (−0.26, 2.26)	0.12
BD + tDCS/BD	1	NR	NR	1.57 (−0.08, 3.22)	0.06
BD + rTMS/BD	1	NR	NR	0.86 (−0.50, 2.22)	0.22
SSA					
BD + Acu/BD	6	0.93	0	−4.38 (−5.32, −3.43)	<0.00001
BD + (BTX-A)/BD	1	NR	NR	−4.80 (−17.11, 7.51)	0.44
BD/CDT	1	NR	NR	−2.90 (−8.44, 2.64)	0.31
rTMS/BD	1	NR	NR	0.72 (−5.93, 7.37)	0.83
BD + rTMS/rTMS	1	NR	NR	−4.17 (−9.16, 0.82)	0.10
BD + EMGBF/BD	1	NR	NR	−2.84 (−6.34, 0.66)	0.11
BD + rTMS/BD	1	NR	NR	−3.45 (−9.38, 2.48)	0.25

VFSS, videofluoroscopic swallowing study; FOIS, Functional Oral Intake Scale; SSA, Standardized Swallowing Assessment; TPRF, tongue pressure resistance feedback; NMES, neuromuscular electrical stimulation; tDCS, transcranial direct current stimulation; rTMS, repetitive transcranial magnetic stimulation; EMGBF, electromyographic biofeedback; BTX-A, botulinum toxin type A; CDT, conventional dysphagia training; BD, balloon dilation; Acu, acupuncture; NR, not reported.

#### 3.4.2 VFSS

Regarding the improvement of VFSS scores, a total of 28 studies involving 11 intervention protocols were included. A fixed-effects model was applied due to low heterogeneity across studies (I^2^ < 50%, *p* ≥ 0.05).

The results indicated the following: balloon dilation combined with acupuncture, balloon dilation combined with TPRF, and TPRF alone were all statistically superior to balloon dilation alone; balloon dilation combined with acupuncture, balloon dilation combined with NMES, and balloon dilation alone were all superior to CDT; balloon dilation combined with TPRF was superior to TPRF alone; balloon dilation combined with NMES was superior to NMES alone; and balloon dilation combined with acupuncture was superior to acupuncture alone. These observed differences reached statistical significance (*p* < 0.05). Detailed outcome data are presented in Table 2, while Supplementary Figure 2 provides comprehensive pairwise meta-analysis findings.

#### 3.4.3 FOIS

Regarding the improvement of FOIS scores, a total of 16 studies involving 11 intervention protocols were included. Statistical analysis indicated substantial heterogeneity among studies comparing balloon dilation combined with NMES versus CDT. The heterogeneity test yielded the following results (I^2^ = 67%, *p* = 0.05). Following sensitivity analysis, the observed heterogeneity decreased, leading to the adoption of a random-effects model. The heterogeneity of comparisons among the remaining groups was relatively low (I^2^ < 50%, *p* ≥ 0.05); therefore, a fixed-effects model was employed for the analysis.

The findings demonstrated that in terms of FOIS score improvement: balloon dilation combined with acupuncture, balloon dilation combined with BTX-A, balloon dilation combined with TPRF, and TPRF alone were statistically superior to balloon dilation alone; balloon dilation combined with NMES and balloon dilation alone were superior to CDT; balloon dilation combined with TPRF was superior to TPRF alone; and balloon dilation combined with NMES was superior to NMES alone. These observed differences reached statistical significance (*p* < 0.05). Detailed findings are presented in Table 2, with additional pairwise meta-analysis data available in Supplementary Figure 3.

#### 3.4.4 SSA

In studies focusing on reducing SSA scores, a total of 12 studies were included, encompassing 7 distinct intervention protocols. Given minimal heterogeneity observed across studies (I^2^ < 50%, *p* ≥ 0.05), a fixed-effects model was employed.

The results demonstrated that, in terms of reducing SSA scores, balloon dilation combined with acupuncture was statistically superior to balloon dilation alone, with this difference reaching statistical significance (*p* < 0.05). Comprehensive data are presented in Table 2, while detailed pairwise meta-analysis outcomes appear in Supplementary Figure 4.

### 3.5 Network meta-analysis

#### 3.5.1 Network evidence graph

**Figure 3:**
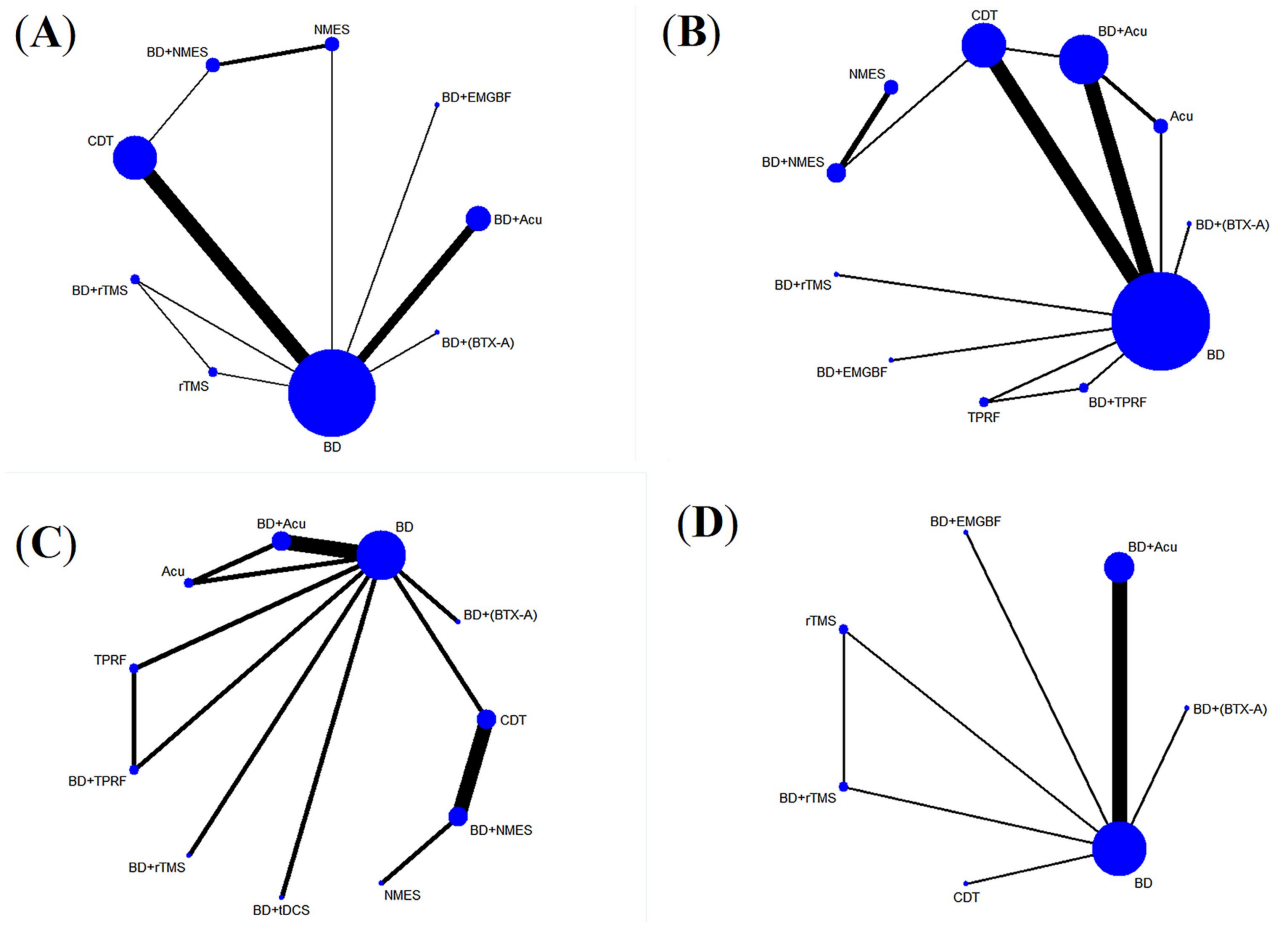
Network meta-analysis diagrams of eligible comparisons: **(A)** effective rate; **(B)** VFSS; **(C)** FOIS; **(D)** SSA.

The graphical representations of network evidence for each outcome indicator are presented in Figure 3. Each data point on the graph corresponds to a distinct therapeutic approach. The size of these points reflect the sample size in each respective clinical trial. Interconnecting lines between data points indicate the number of head-to-head comparative studies conducted between treatment modalities, with thicker lines representing more frequent direct comparisons.

#### 3.5.2 Transitivity assessment

To assess potential systematic bias in indirect comparisons, this study performed transitivity verification for four key effect modifiers (mean age, gender ratio, stroke duration, and intervention duration) that influence the outcomes. Box plots were used to present the distribution balance of each covariate, as detailed in Supplementary Figure 5. The box plots based on these effect modifiers showed that there was obvious overall overlap in the covariate distributions across different intervention comparisons, suggesting that there were no systematic differences in the key effect modifiers and the transitivity assumption was valid.

#### 3.5.3 Results of the inconsistency test

##### 3.5.3.1 Effective rate

Regarding the effective rate outcome indicator, the overall inconsistency test yielded *p* = 0.7948 (*p* > 0.05, Supplementary Figure 6a). The local inconsistency test using the node-splitting method indicated that all pairwise comparison results showed *p* > 0.05 (Supplementary Figure 6b). The loop inconsistency test result was *p* = 0.795 (*p* > 0.05), with a 95% confidence interval of (0.00, 3.25), which included zero (Supplementary Figure 6c). These results demonstrated good consistency between direct and indirect evidence, thereby supporting the use of a consistency model for subsequent analytical procedures.

##### 3.5.3.2 VFSS

Regarding the outcome indicators of VFSS, the global inconsistency test yielded *p* = 0.7014 (*p* > 0.05, Supplementary Figure 7a). Subsequent local inconsistency analysis employing the node-splitting method revealed that all pairwise comparisons had *p* > 0.05 (Supplementary Figure 7b). Loop-specific inconsistency tests produced *p*-values of 0.606 and 0.696 (both *p* > 0.05), with 95% confidence intervals of (0.00, 2.53) and (0.00, 1.37), respectively. Notably, both confidence intervals included zero (Supplementary Figure 7c). These results collectively demonstrated good consistency between direct and indirect evidence, thereby validating the use of a consistency model for subsequent statistical inference.

##### 3.5.3.3 FOIS

Regarding the outcome indicators of FOIS, the overall inconsistency test yielded *p* = 0.9825 (*p* > 0.05, Supplementary Figure 8a). The local inconsistency test conducted via the node-splitting method revealed that all pairwise comparisons had *p* > 0.05 (Supplementary Figure 8b). The results of the loop inconsistency test were *p* = 0.981 (*p* > 0.05), with a 95% confidence interval of (0.00, 1.92), which included zero (Supplementary Figure 8c). These results demonstrated good consistency between direct and indirect evidence. Therefore, a consistency model was adopted for analysis.

##### 3.5.3.4 SSA

Regarding the outcome indicators of SSA, although a closed loop was formed, all interventions within this loop were derived from the same three-arm trial. Consequently, the node-splitting method was employed to assess inconsistency within the closed loop. Statistical analysis indicated that all pairwise comparisons yielded *p* > 0.05 (Supplementary Figure 9), demonstrating no significant inconsistency.

#### 3.5.4 Comparison results of cumulative probability ranking for the included studies

##### 3.5.4.1 Effective rate

**Figure 4:**
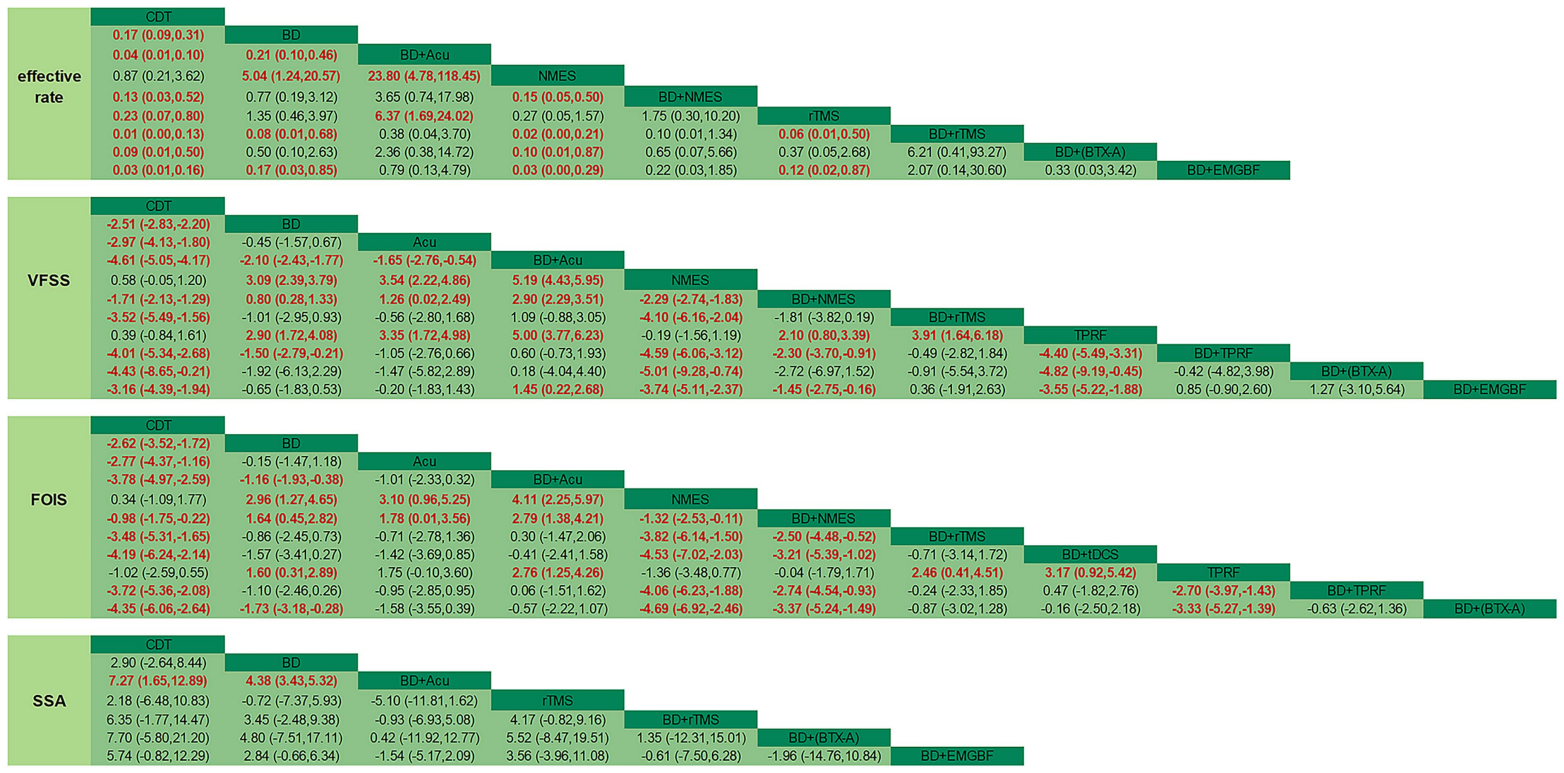
Network meta-analysis of head-to-head comparisons. Red and bold numbers are statistically significant.

Regarding the effective rate outcome indicator, a total of 18 studies were included, encompassing 9 distinct types of intervention measures. The results indicated that all interventions except NMES showed statistically significant differences compared with CDT in terms of effective rate scores. The outcomes of pairwise comparisons are presented in detail in Figure 4.

**Figure 5:**
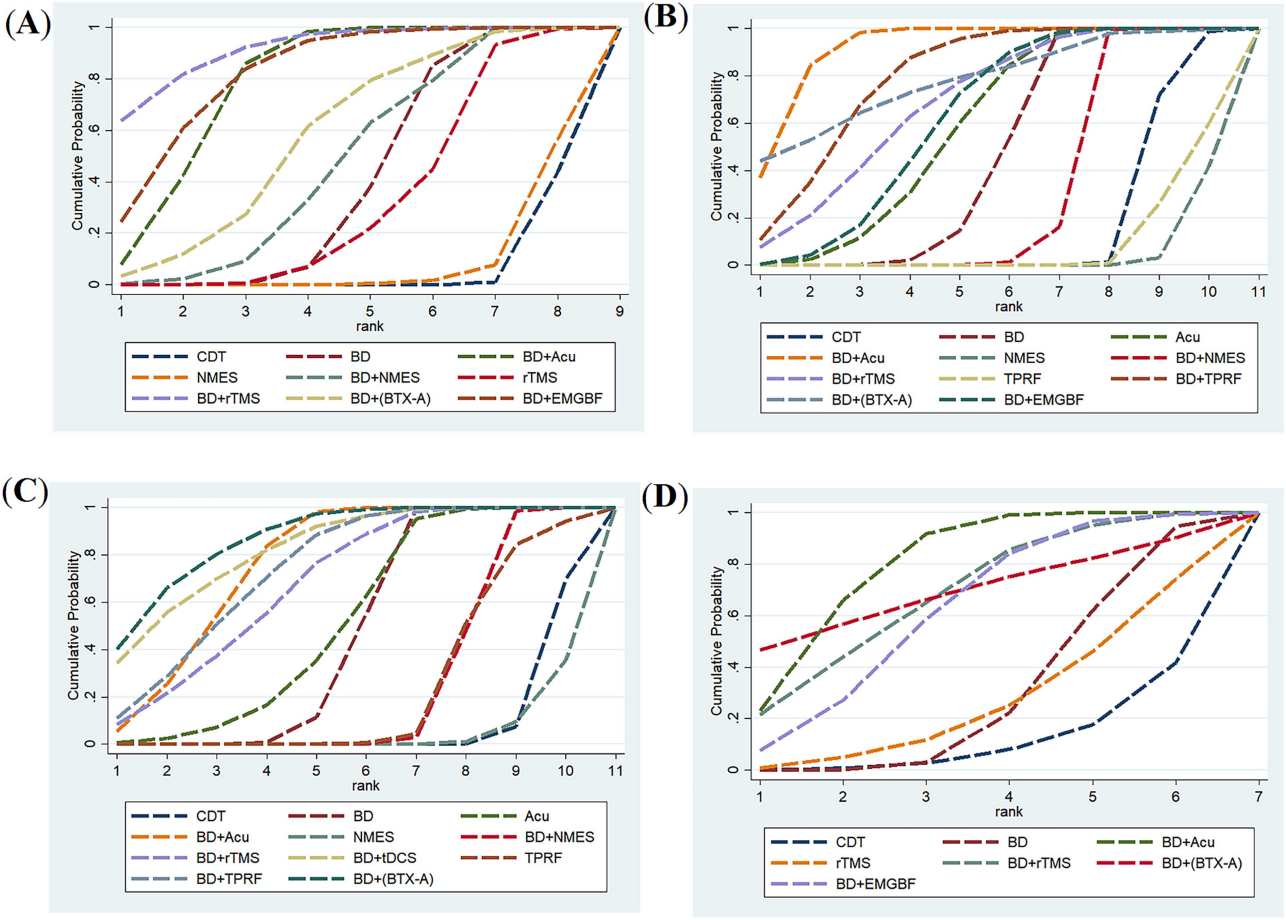
Surface under the cumulative ranking curve (SUCRA) analysis for assessing the relative effectiveness of interventions. SUCRA values, depicted as the area under the curve, provide a probabilistic ranking of interventions, with larger values indicating a greater likelihood of being among the most effective. **(A)** Effective rate; **(B)** VFSS; **(C)** FOIS; **(D)** SSA.

The surface under the cumulative ranking curve (SUCRA) values suggested that interventions ranked in descending order as follows: balloon dilation combined with rTMS (91.8%) > balloon dilation combined with EMGBF (82.8%) > balloon dilation combined with acupuncture (79.4%) > balloon dilation combined with BTX-A (58.9%) > balloon dilation combined with NMES (48.5%) > balloon dilation (41.3%) > rTMS (33.4%) > NMES (8.4%) > CDT (5.6%). Based on existing evidence, balloon dilation combined with rTMS may be the most effective intervention in terms of improving the effective rate. Cumulative probability comparison results are shown in Figure 5.

##### 3.5.4.2 VFSS

Regarding the outcome indicators of VFSS, a total of 28 studies were included, encompassing 11 distinct types of intervention measures. The results indicated that, with the exception of NMES and TPRF, all other intervention methods showed statistically significant differences in VFSS scores compared with the CDT group. The outcomes of pairwise comparisons are presented in detail in Figure 4.

The surface under the cumulative ranking curve (SUCRA) values suggested that interventions ranked in descending order as follows: balloon dilation combined with acupuncture (92.0%) > balloon dilation combined with TPRF (79.6%) > balloon dilation combined with BTX-A (78.4%) > balloon dilation combined with rTMS (69.4%) > balloon dilation combined with EMGBF (62.7%) > acupuncture (58.8%) > balloon dilation (47.0%) > balloon dilation combined with NMES (31.7%) > CDT (17.2%) > TPRF (8.7%) > NMES (4.5%). Based on existing evidence, balloon dilation combined with acupuncture may be the most effective intervention in terms of improving VFSS scores. Cumulative probability comparison results are shown in Figure 5.

##### 3.5.4.3 FOIS

Regarding the outcome indicators of FOIS, a total of 16 studies were included, encompassing 11 distinct types of intervention measures. The results indicated that, with the exception of NMES and TPRF, all other intervention methods exhibit statistically significant differences in FOIS scores compared with the CDT group. The outcomes of pairwise comparisons are detailed in Figure 4.

The surface under the cumulative ranking curve (SUCRA) values suggested that interventions ranked in descending order as follows: balloon dilation combined with BTX-A (87.4%) > balloon dilation combined with tDCS (83.0%) > balloon dilation combined with acupuncture (76.8%) > balloon dilation combined with TPRF (74.6%) > balloon dilation combined with rTMS (68.6%) > acupuncture (52.0%) > balloon dilation (46.7%) > balloon dilation combined with NMES (25.0%) > TPRF (23.6%) > CDT (7.8%) > NMES (4.6%). Based on existing evidence, balloon dilation combined with BTX-A may be the most effective intervention in terms of improving FOIS scores. Cumulative probability comparison results are shown in Figure 5.

##### 3.5.4.4 SSA

Regarding the outcome indicators of SSA, a total of 12 studies were included, encompassing 7 distinct types of intervention measures. Balloon dilation combined with acupuncture demonstrated statistically significant differences compared with both CDT and balloon dilation alone. The outcomes of pairwise comparisons are detailed in Figure 4.

The surface under the cumulative ranking curve (SUCRA) values suggested that interventions ranked in descending order as follows: balloon dilation combined with acupuncture (80.0%) > balloon dilation combined with BTX-A (69.5%) > balloon dilation combined with rTMS (68.5%) > balloon dilation combined with EMGBF (62.4%) > balloon dilation (30.4%) > rTMS (27.3%) > CDT (11.9%). Based on existing evidence, balloon dilation combined with acupuncture may be the most effective intervention in terms of improving SSA scores. Cumulative probability comparison results are shown in Figure 5.

### 3.6 Adverse reaction

Among all included studies, a total of six reported adverse events. Specifically, three studies reported no adverse reactions or complications during the treatment period. For detailed adverse events documented in the remaining three studies, please refer to Supplementary Table 15. Owing to heterogeneity in outcome assessment criteria across studies, only descriptive analysis was performed in this review.

### 3.7 Publication bias

**Figure 6:**
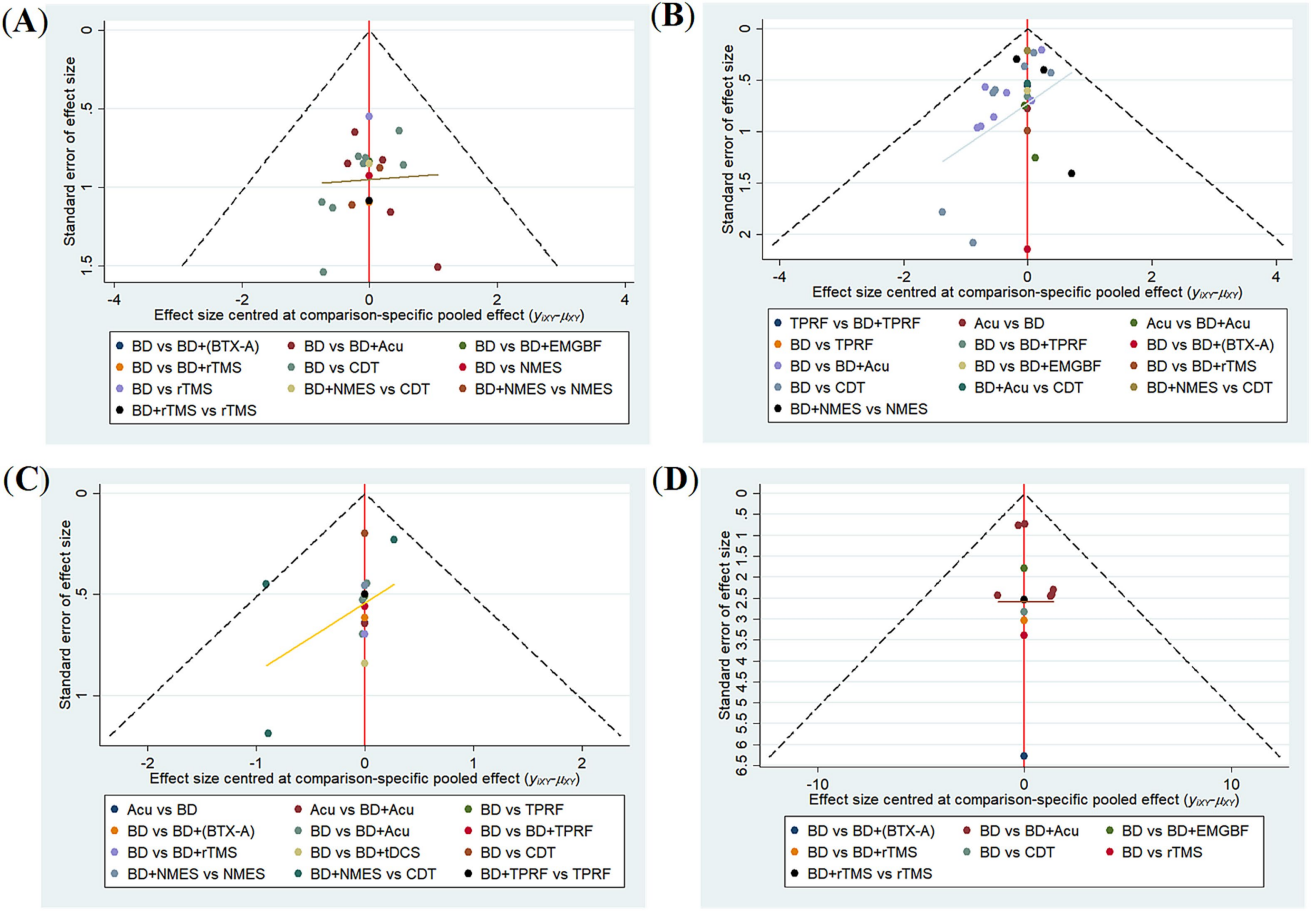
Funnel plot of publication bias. **(A)** Effective rate; **(B)** VFSS; **(C)** FOIS; **(D)** SSA.

The funnel plots for each indicator are shown in Figure 6.

The funnel plot for the effective rate showed an asymmetrical distribution, and Egger’s test results (*p* = 0.006 < 0.05, Supplementary Figure 10) suggested the possible existence of a small-study effect and publication bias. After bias correction using the trim-and-fill method, although the results changed, the differences did not reach statistical significance (Supplementary Figure 11).

The funnel plots for the VFSS scores and FOIS scores showed approximate symmetry on both sides. The results of Egger’s test were *p* = 0.450 > 0.05 and *p* = 0.262 > 0.05, respectively (Supplementary Figure 12), indicating no significant small-study effect or publication bias.

In the funnel plot for the SSA score, the distribution of scatter points was basically symmetric. However, Egger’s test result (*p* = 0.046 < 0.05, Supplementary Figure 13) suggested the possible existence of a slight small-study effect and publication bias. After correction using the trim-and-fill method, although the results showed slight changes, the differences did not reach statistical significance (Supplementary Figure 14).

## 4 Discussion

This study introduces a novel application of network meta-analysis to systematically compare the efficacy of balloon dilation, acupuncture, NMES, rTMS, tDCS, TPRF, BTX-A, EMGBF, and their combined regimens for post-stroke CPA. The analysis results indicate that the efficacy of different interventions varies across outcome indicators. In terms of improving VFSS scores and reducing SSA scores, balloon dilation combined with acupuncture may be the optimal intervention. In terms of improving FOIS scores, balloon dilation combined with BTX-A may be the optimal intervention. In terms of improving the effective rate, balloon dilation combined with rTMS may be the optimal intervention.

From the perspective of clinical relevance in our findings, although statistically significant differences were observed among various interventions in terms of the effective rate, VFSS, FOIS, and SSA outcomes, most of these differences showed small effect sizes. Moreover, given the absence of established minimal clinically important difference (MCID) thresholds for the aforementioned outcome measures, the results of this study only indicate relative advantages among interventions and do not reflect clinically meaningful differences. The actual improvement in swallowing function conferred by these interventions still requires further clinical validation, and the certainty of the current evidence is limited by the small sample sizes and potential risk of bias in the included studies.

The underlying mechanism by which the aforementioned interventions improve swallowing function may be associated with the following pathophysiological pathways. As the core indicators for assessing swallowing physiological function and aspiration risk, VFSS and SSA show that balloon dilation combined with acupuncture demonstrates a superior improvement effect. Previous research indicates that the principal mechanisms of balloon dilation for post-stroke CPA involve the following three theoretical frameworks: biomechanical mechanisms, central nervous system regulation mechanisms, and cerebral cortical plasticity mechanisms (3). Balloon dilation directly acts on the upper esophageal sphincter through mechanical forces, addressing issues of anatomical stenosis or incomplete relaxation. Simultaneously, during the dilation process of the cricopharyngeal muscle, it provides sensory feedback to the swallowing control centers in the brain, thereby reinforcing impaired cortical and subcortical connections, facilitating neural remodeling, and improving swallowing function (47). The potential mechanism of acupuncture in treating dysphagia may involve not only the stimulation of muscle groups associated with swallowing function but also, based on holistic syndrome differentiation and treatment, the enhancement of blood circulation in the cerebral cortex’s motor functional areas, the promotion of cerebral energy metabolism, and the activation of specific cortical motor regions, thereby facilitating brain function remodeling (48). Furthermore, acupuncture can stimulate the glossopharyngeal, hypoglossal, and vagus nerves, modulate the excitability of swallowing-related central nervous system components, enhance neural plasticity, and consequently promote the recovery of swallowing ability (49). The combination of these mechanisms may yield a synergistic effect of physical dilation and neuromuscular regulation. A meta-analysis encompassing 617 patients demonstrated that balloon dilation combined with acupuncture significantly improves post-stroke CPA (50), which is consistent with the findings of our study.

FOIS serves as a core outcome for evaluating patients’ actual eating ability and dietary intake level, and balloon dilation combined with BTX-A demonstrates a superior improvement effect. BTX-A, a neurotoxic protein, inhibits neuromuscular transmission by blocking acetylcholine release from presynaptic cholinergic nerve terminals. BTX-A injection has demonstrated efficacy in treating various spasticity-related disorders (51). Administration of BTX-A into the cricopharyngeal muscle reduces the resting pressure of the upper esophageal sphincter and prolongs its relaxation duration, thereby facilitating swallowing function recovery (52, 53). The therapeutic effect of BTX-A typically persists for 3 to 6 months, with possible extension to 1 year or longer (54). Currently, no definitive guidelines or consensus exist regarding indications and contraindications for BTX-A therapy in cricopharyngeal dysfunction. It is primarily employed when rehabilitation training and balloon dilation yield suboptimal outcomes (55). BTX-A induces chemical relaxation of the cricopharyngeal muscle, whereas balloon dilation mechanically expands the upper esophageal sphincter through physical force, thereby addressing issues of anatomical stenosis or inadequate relaxation. The combined application of these two modalities represents one of the effective approaches for treating swallowing disorders resulting from post-stroke CPA and holds value for clinical dissemination.

With effective rate as the comprehensive clinical evaluation index, balloon dilation combined with rTMS exhibited a better improvement effect. rTMS constitutes an advanced non-invasive neurological rehabilitation technique based on the principle of electromagnetic induction. It modulates cortical excitability, influences cerebral physiological functions by inducing changes in neuronal potentials, and offers advantages including painless application, notable therapeutic effects, and minimal adverse reactions, which have led to its widespread adoption in clinical practice (56). The balloon dilation technique effectively stimulates and enhances the functionality of swallowing-related muscle groups through mechanical dilation. rTMS directly targets the cerebral cortex; by regulating cortical excitability and enhancing or inhibiting the compensatory mechanisms of the central nervous system, it facilitates cortical reorganization, thereby improving swallowing function (57). Furthermore, post-stroke dysphagia in clinical settings often presents multiple complex issues, such as concurrent reduction in pharyngeal muscle strength, inadequate laryngeal elevation, and impaired cricopharyngeal muscle activity. Consequently, combined central and peripheral interventions can yield superior therapeutic outcomes.

However, this study must acknowledge several limitations. First, the primary outcome measure “effective rate” employed in this research is not an internationally standardized metric, and the heterogeneous definitions applied across the included original studies may have affected the accuracy of both the ranking of efficacy outcomes and the estimation of effect sizes. Second, although transitivity assessment revealed no systematic differences in key effect modifiers (mean age, gender ratio, stroke duration and intervention duration), unmeasured effect modifiers still exist, including stroke severity, disease stage, as well as differences in stimulation parameters and acupuncture protocols. These factors may introduce potential bias into indirect comparison results and intervention ranking. Due to the limited number and sample size of included studies, we were unable to perform subgroup analyses or meta-regression to quantitatively explore the impact of these sources of heterogeneity. Future large-scale, rigorously designed clinical trials with standardized interventions and participant characteristics are warranted to address these limitations. Third, some of the included studies were limited by small sample sizes, inadequate allocation concealment, and insufficient blinding, raising concerns regarding the risk of bias. In addition, both the response rate and SSA outcome measures may be subject to publication bias, further diminishing the certainty of the evidence. Fourth, head-to-head randomized controlled trials directly comparing certain combination therapies are currently lacking, and the existing rankings largely depend on indirect comparisons, which entail comparatively limited inferential strength. Fifth, the present analysis focuses predominantly on short-term efficacy indicators, while long-term benefits and optimal treatment durations remain undetermined. Lastly, although a systematic and comprehensive literature search was conducted, the majority of included studies were published in Chinese, with a limited number of eligible English-language publications, suggesting possible language selection bias. This limitation may constrain the generalizability of the findings. Future research should involve international, multicenter, large-scale, high-quality randomized controlled trials to directly compare the clinical efficacy of various combination therapies.

## 5 Conclusion

In summary, this network meta-analysis demonstrates that integrated interventions centered on balloon dilation exhibit superior overall efficacy compared to monotherapy in the management of post-stroke CPA, although the relative advantages vary across different combination strategies. Based on probability ranking results, balloon dilation combined with rTMS may be superior in improving the effective rate. Balloon dilation combined with acupuncture shows relatively greater improvement in VFSS scores and reduction in SSA scores, whereas balloon dilation combined with BTX-A demonstrates relative superiority in enhancing FOIS scores. It should be emphasized that these probability rankings reflect relative advantages rather than clinically significant differences. Due to limitations such as small sample sizes in included studies, inconsistent definitions of certain outcome measures, potential publication bias, and a lack of direct comparison evidence among some combination therapies, the current findings should be interpreted with caution. Further high-quality studies are warranted to validate these results.

## Data Availability

The original contributions presented in the study are included in the article/Supplementary material, further inquiries can be directed to the corresponding author.
