# Supplementary material for: Comparative efficacy of different interventions for post-stroke cricopharyngeal achalasia: a systematic review and network meta-analysis
Source: Front Neurol. 2026 Apr 10;17:1802305. doi: 10.3389/fneur.2026.1802305 (PMC13105938; doi:10.3389/fneur.2026.1802305)
Supplement: Supplementary file 1 [file Table_1.DOCX]

Supplementary Material

# Supplementary **Tables**

**Supplementary Table 1.** the search strategy of PubMed.

| #1 | "Stroke"[Mesh] |
| --- | --- |
| #2 | (((((((((((((((((((((((((((Strokes[Title/Abstract]) OR (Cerebrovascular Accident[Title/Abstract])) OR (Cerebrovascular Accidents[Title/Abstract])) OR (Cerebral Stroke[Title/Abstract])) OR (Cerebral Strokes[Title/Abstract])) OR (Stroke, Cerebral[Title/Abstract])) OR (Strokes, Cerebral[Title/Abstract])) OR (Cerebrovascular Apoplexy[Title/Abstract])) OR (Apoplexy, Cerebrovascular[Title/Abstract])) OR (Vascular Accident, Brain[Title/Abstract])) OR (Brain Vascular Accident[Title/Abstract])) OR (Brain Vascular Accidents[Title/Abstract])) OR (Vascular Accidents, Brain[Title/Abstract])) OR (Cerebrovascular Stroke[Title/Abstract])) OR (Cerebrovascular Strokes[Title/Abstract])) OR (Stroke, Cerebrovascular[Title/Abstract])) OR (Strokes, Cerebrovascular[Title/Abstract])) OR (Apoplexy[Title/Abstract])) OR (CVA (Cerebrovascular Accident[Title/Abstract]))) OR (CVAs (Cerebrovascular Accident[Title/Abstract]))) OR (Stroke, Acute[Title/Abstract])) OR (Acute Stroke[Title/Abstract])) OR (Acute Strokes[Title/Abstract])) OR (Strokes, Acute[Title/Abstract])) OR (Cerebrovascular Accident, Acute[Title/Abstract])) OR (Acute Cerebrovascular Accident[Title/Abstract])) OR (Acute Cerebrovascular Accidents[Title/Abstract])) OR (Cerebrovascular Accidents, Acute[Title/Abstract]) |
| #3 | #1 OR #2 |
| #4 | ((((cricopharyngeal achalasia[Title/Abstract]) OR (cricopharyngeal muscle[Title/Abstract])) OR (achalasia[Title/Abstract])) OR (cricopharyngeal dysfunction[Title/Abstract])) OR (Achalasia of the upper esophageal sphincter[Title/Abstract]) |
| #5 | #3 AND #4 |
| #6 | ((((((((((((((((((((((((((((((((((Magnetic Stimulations, Transcranial[Title/Abstract]) OR (Magnetic Stimulation, Transcranial[Title/Abstract])) OR (Stimulations, Transcranial Magnetic[Title/Abstract])) OR (Stimulation, Transcranial Magnetic[Title/Abstract])) OR (Transcranial Magnetic Stimulations[Title/Abstract])) OR (Transcranial Magnetic Stimulation, Paired Pulse[Title/Abstract])) OR (Transcranial Magnetic Stimulation, Repetitive[Title/Abstract])) OR (Transcranial Magnetic Stimulation, Single Pulse[Title/Abstract])) OR (tDCS[Title/Abstract])) OR (Anodal Stimulation Transcranial Direct Current Stimulation[Title/Abstract])) OR (Cathodal Stimulation Transcranial Direct Current Stimulation[Title/Abstract])) OR (Neuromuscular Electrical Stimulation[Title/Abstract])) OR (sensory neuromuscular electrical Stimulation[Title/Abstract])) OR (Surface Neuro Muscular Electrical Stimulation[Title/Abstract])) OR (Therapeutic Electrical Stimulation[Title/Abstract])) OR (Therapeutic Electric Stimulation[Title/Abstract])) OR (Electrotherapy[Title/Abstract])) OR (NMES[Title/Abstract])) OR (sNMES[Title/Abstract])) OR (acupuncture[Title/Abstract])) OR (Electroacupuncture[Title/Abstract])) OR (Catheter balloon dilatation[Title/Abstract])) OR (balloon dilatation[Title/Abstract])) OR (Botulinum toxin injection[Title/Abstract])) OR (electromyographic biofeedback[Title/Abstract])) OR (electromyography biofeedback[Title/Abstract])) OR (Surface electromyography biofeedback[Title/Abstract])) OR (Tongue pressure resistance feedback[Title/Abstract])) OR (tongue pressure feedback[Title/Abstract])) OR (Conventional Dysphagia Therapy[Title/Abstract])) OR (Conventional swallowing training[Title/Abstract])) OR (wallowing training[Title/Abstract])) OR (swallowing exercise[Title/Abstract])) OR (swallowing therapy[Title/Abstract])) OR (Swallowing rehabilitation therapy[Title/Abstract]) |
| #7 | (randomized controlled trial [Publication Type] OR randomized [Title/Abstract] OR placebo[Title/Abstract]) |
| #8 | #5 AND #6 AND #7 |

**Supplementary Table 2.** the search strategy of Embase.

| #1 | 'cerebrovascular accident'/exp |
| --- | --- |
| #2 | 'strokes':ab,ti OR 'cerebrovascular accident':ab,ti OR 'cerebrovascular accidents':ab,ti OR 'cerebral stroke':ab,ti OR 'cerebral strokes':ab,ti OR 'Stroke, Cerebral':ab,ti OR 'Strokes, Cerebral':ab,ti OR 'Cerebrovascular Apoplexy':ab,ti OR 'Apoplexy, Cerebrovascular':ab,ti OR 'Vascular Accident, Brain':ab,ti OR 'Brain Vascular Accident':ab,ti OR 'Brain Vascular Accidents':ab,ti OR 'Vascular Accidents, Brain':ab,ti OR 'Cerebrovascular Stroke':ab,ti OR 'Cerebrovascular Strokes':ab,ti OR 'Stroke, Cerebrovascular':ab,ti OR 'Strokes, Cerebrovascular':ab,ti OR 'Apoplexy':ab,ti OR 'CVA (Cerebrovascular Accident)':ab,ti OR 'CVAs (Cerebrovascular Accident)':ab,ti OR 'Stroke, Acute':ab,ti OR 'Acute Stroke':ab,ti OR 'Acute Strokes':ab,ti OR 'Strokes, Acute':ab,ti OR 'Cerebrovascular Accident, Acute':ab,ti OR 'Acute Cerebrovascular Accident':ab,ti OR 'Acute Cerebrovascular Accidents':ab,ti OR 'Cerebrovascular Accidents, Acute':ab,ti |
| #3 | #1 OR #2 |
| #4 | 'cricopharyngeal achalasia':ab,ti OR 'cricopharyngeal muscle':ab,ti OR 'achalasia':ab,ti OR 'cricopharyngeal dysfunction':ab,ti OR 'achalasia of the upper esophageal sphincter':ab,ti |
| #5 | #3 AND #4 |
| #6 | 'transcranial magnetic stimulation'/exp OR 'transcranial magnetic stimulation' |
| #7 | 'Transcranial Direct Current Stimulation'/exp OR 'Transcranial Direct Current Stimulation' |
| #8 | 'electrostimulation'/exp OR 'electrostimulation' |
| #9 | 'acupuncture'/exp OR 'acupuncture' |
| #10 | 'electroacupuncture'/exp OR 'electroacupuncture' |
| #11 | 'magnetic stimulations, transcranial':ab,ti OR 'magnetic stimulation, transcranial':ab,ti OR 'stimulations, transcranial magnetic':ab,ti OR 'stimulation, transcranial magnetic':ab,ti OR 'transcranial magnetic stimulations':ab,ti OR 'transcranial magnetic stimulation, paired pulse':ab,ti OR 'transcranial magnetic stimulation, repetitive':ab,ti OR 'transcranial magnetic stimulation, single pulse':ab,ti OR 'tDCS':ab,ti OR 'Anodal Stimulation Transcranial Direct Current Stimulation':ab,ti OR 'Cathodal Stimulation Transcranial Direct Current Stimulation':ab,ti OR 'neuromuscular electrical stimulation':ab,ti OR 'sensory neuromuscular electrical stimulation':ab,ti OR 'surface neuro muscular electrical stimulation':ab,ti OR 'therapeutic electrical stimulation':ab,ti OR 'therapeutic electric stimulation':ab,ti OR 'electrotherapy':ab,ti OR 'nmes':ab,ti OR 'snmes':ab,ti OR 'acupuncture':ab,ti OR 'electroacupuncture':ab,ti OR 'catheter balloon dilatation':ab,ti OR 'balloon dilatation':ab,ti OR 'botulinum toxin injection':ab,ti OR 'electromyographic biofeedback':ab,ti OR 'electromyography biofeedback':ab,ti OR 'surface electromyography biofeedback':ab,ti OR 'tongue pressure resistance feedback':ab,ti OR 'tongue pressure feedback':ab,ti OR 'conventional dysphagia therapy':ab,ti OR 'conventional swallowing training':ab,ti OR 'swallowing training':ab,ti OR 'swallowing exercise':ab,ti OR 'swallowing therapy':ab,ti OR 'swallowing rehabilitation therapy':ab,ti |
| #12 | #6 OR #7 OR #8 OR #9 OR #10 OR #11 |
| #13 | 'randomized controlled trial':ab,ti OR 'randomized ':ab,ti OR 'placebo':ab,ti OR 'RCT':ab,ti |
| #14 | #5 AND #12 AND #13 |

**Supplementary Table 3.** the search strategy of Web of Science.

| #1 | TS=(stroke or strokes or Cerebrovascular Accident or Cerebrovascular Accidents or Cerebral Stroke or Cerebral Strokes or Stroke, Cerebral or Strokes, Cerebral or Cerebrovascular Apoplexy or Apoplexy, Cerebrovascular or Vascular Accident, Brain or Brain Vascular Accident or Brain Vascular Accidents or Vascular Accidents, Brain or Cerebrovascular Stroke or Cerebrovascular Strokes or Stroke, Cerebrovascular or Strokes, Cerebrovascular or Apoplexy or CVA (Cerebrovascular Accident) or CVAs (Cerebrovascular Accident) or Stroke, Acute or Acute Stroke or Acute Strokes or Strokes, Acute or Cerebrovascular Accident, Acute or Acute Cerebrovascular Accidents or Cerebrovascular Accidents, Acute) |
| --- | --- |
| #2 | TS=(cricopharyngeal achalasia or cricopharyngeal muscle or achalasia or cricopharyngeal dysfunction or Achalasia of the upper esophageal sphincter) |
| #3 | #1 AND #2 |
| #4 | TS=(Transcranial Magnetic Stimulation or Magnetic Stimulations, Transcranial or Magnetic Stimulation, Transcranial or Stimulations, Transcranial Magnetic or Stimulation, Transcranial Magnetic or Transcranial Magnetic Stimulations or Transcranial Magnetic Stimulation, Paired Pulse or Transcranial Magnetic Stimulation, Repetitive or Transcranial Magnetic Stimulation, Single Pulse or Transcranial Direct Current Stimulation or tDCS or Anodal Stimulation Transcranial Direct Current Stimulation or Cathodal Stimulation Transcranial Direct Current Stimulation or Electric Stimulation or Neuromuscular Electrical Stimulation or sensory neuromuscular electrical Stimulation or Surface Neuro Muscular Electrical Stimulation or Therapeutic Electrical Stimulation or Therapeutic Electric Stimulation or Electrotherapy or NMES or sNMES or acupuncture or Electroacupuncture or Catheter balloon dilatation or balloon dilatation or Botulinum toxin injection or electromyographic biofeedback or electromyography biofeedback or Surface electromyography biofeedback or Tongue pressure resistance feedback or tongue pressure feedback or Conventional Dysphagia Therapy or Conventional swallowing training or swallowing training or swallowing exercise or swallowing therapy or Swallowing rehabilitation therapy) |
| #5 | AB=(randomized controlled trial or randomized or placebo or RCT) |
| #6 | #3 AND #4 AND #5 |

**Supplementary Table 4.** the search strategy of Cochrane Library.

| #1 | MeSH descriptor: [Stroke] explode all trees |
| --- | --- |
| #2 | (strokes):ti,ab,kw OR (cerebrovascular accident):ti,ab,kw OR (Cerebrovascular Accidents):ti,ab,kw OR (Cerebral Stroke):ti,ab,kw OR (Cerebral Strokes):ti,ab,kw OR (Stroke, Cerebral):ti,ab,kw OR (Strokes, Cerebral):ti,ab,kw OR (Cerebrovascular Apoplexy):ti,ab,kw OR (Apoplexy, Cerebrovascular):ti,ab,kw OR (Vascular Accident, Brain):ti,ab,kw OR (Brain Vascular Accident):ti,ab,kw OR (Brain Vascular Accidents):ti,ab,kw OR (Vascular Accidents, Brain):ti,ab,kw OR (Cerebrovascular Stroke):ti,ab,kw OR (Cerebrovascular Strokes):ti,ab,kw OR (Stroke, Cerebrovascular):ti,ab,kw OR (Stroke, Cerebrovascular):ti,ab,kw OR (Strokes, Cerebrovascular):ti,ab,kw OR (Apoplexy):ti,ab,kw OR (CVA (Cerebrovascular Accident)):ti,ab,kw OR (CVAs (Cerebrovascular Accident)):ti,ab,kw OR (Stroke, Acute):ti,ab,kw OR (Acute Stroke):ti,ab,kw OR (Acute Strokes):ti,ab,kw OR (Strokes, Acute):ti,ab,kw OR (Cerebrovascular Accident, Acute):ti,ab,kw OR (Acute Cerebrovascular Accidents):ti,ab,kw OR (Cerebrovascular Accidents, Acute):ti,ab,kw |
| #3 | #1 OR #2 |
| #4 | (cricopharyngeal achalasia):ti,ab,kw OR (cricopharyngeal muscle):ti,ab,kw OR (achalasia):ti,ab,kw OR (cricopharyngeal dysfunction):ti,ab,kw OR (Achalasia of the upper esophageal sphincter):ti,ab,kw |
| #5 | #3 AND #4 |
| #6 | MeSH descriptor: [Transcranial Magnetic Stimulation] explode all trees |
| #7 | MeSH descriptor: [Transcranial Direct Current Stimulation] explode all trees |
| #8 | MeSH descriptor: [Electric Stimulation] explode all trees |
| #9 | MeSH descriptor: [Acupuncture] explode all trees |
| #10 | MeSH descriptor: [Electroacupuncture] explode all trees |
| #11 | (Magnetic Stimulations, Transcranial):ti,ab,kw OR (Magnetic Stimulation, Transcranial):ti,ab,kw OR (Stimulations, Transcranial Magnetic):ti,ab,kw OR (Stimulation, Transcranial Magnetic):ti,ab,kw OR (Transcranial Magnetic Stimulations):ti,ab,kw OR (Transcranial Magnetic Stimulation, Paired Pulse):ti,ab,kw OR (Transcranial Magnetic Stimulation, Repetitive):ti,ab,kw OR (Transcranial Magnetic Stimulation, Single Pulse):ti,ab,kw OR (tDCS):ti,ab,kw OR (Anodal Stimulation Transcranial Direct Current Stimulation):ti,ab,kw OR (Cathodal Stimulation Transcranial Direct Current Stimulation):ti,ab,kw OR (Neuromuscular Electrical Stimulation):ti,ab,kw OR (sensory neuromuscular electrical Stimulation):ti,ab,kw OR (Surface Neuro Muscular Electrical Stimulation):ti,ab,kw OR (Therapeutic Electrical Stimulation):ti,ab,kw OR (Therapeutic Electric Stimulation):ti,ab,kw OR (Electrotherapy):ti,ab,kw OR (NMES):ti,ab,kw OR (sNMES):ti,ab,kw OR (acupuncture):ti,ab,kw OR (Electroacupuncture):ti,ab,kw OR (Catheter balloon dilatation):ti,ab,kw OR (balloon dilatation):ti,ab,kw OR (Botulinum toxin injection):ti,ab,kw OR (electromyographic biofeedback):ti,ab,kw OR (electromyography biofeedback):ti,ab,kw OR (Surface electromyography biofeedback):ti,ab,kw OR (Tongue pressure resistance feedback):ti,ab,kw OR (tongue pressure feedback):ti,ab,kw OR (Conventional Dysphagia Therapy):ti,ab,kw OR (Conventional swallowing training):ti,ab,kw OR (swallowing training):ti,ab,kw OR (swallowing exercise):ti,ab,kw OR (swallowing therapy):ti,ab,kw OR (Swallowing rehabilitation therapy):ti,ab,kw |
| #12 | #6 OR #7 OR #8 OR #9 OR #10 OR #11 |
| #13 | (randomized controlled trial):ti,ab,kw OR (randomized):ti,ab,kw OR (placebo):ti,ab,kw OR (RCT):ti,ab,kw |
| #14 | #5 AND #12 AND #13 |

**Supplementary Table 5.** the search strategy of SCOPUS.

| TITLE-ABS-KEY(“stroke” or “strokes” or “Cerebrovascular Accident” or “Cerebrovascular Accidents” or “Cerebral Stroke” or “Cerebral Strokes” or “Stroke, Cerebral” or “Strokes, Cerebral” or “Cerebrovascular Apoplexy” or “Apoplexy, Cerebrovascular” or “Vascular Accident, Brain” or “Brain Vascular Accident” or “Brain Vascular Accidents” or “Vascular Accidents, Brain” or “Cerebrovascular Stroke” or “Cerebrovascular Strokes” or “Stroke, Cerebrovascular” or “Strokes, Cerebrovascular” or “Apoplexy or CVA (Cerebrovascular Accident)” or “CVAs (Cerebrovascular Accident)” or “Stroke, Acute” or “Acute Stroke” or “Acute Strokes” or “Strokes, Acute” or “Cerebrovascular Accident, Acute” or “Acute Cerebrovascular Accidents” or “Cerebrovascular Accidents, Acute”) AND TITLE-ABS-KEY(“cricopharyngeal achalasia” or “cricopharyngeal muscle” or “achalasia” or “cricopharyngeal dysfunction” or “Achalasia of the upper esophageal sphincter”) AND TITLE-ABS-KEY(“Transcranial Magnetic Stimulation” or “Magnetic Stimulations, Transcranial” or “Magnetic Stimulation, Transcranial” or “Stimulations, Transcranial Magnetic” or “Stimulation, Transcranial Magnetic” or “Transcranial Magnetic Stimulations” or “Transcranial Magnetic Stimulation, Paired Pulse” or “Transcranial Magnetic Stimulation, Repetitive” or “Transcranial Magnetic Stimulation, Single Pulse” or “Transcranial Magnetic Stimulations” or “Transcranial Magnetic Stimulation, Paired Pulse” or “Transcranial Magnetic Stimulation, Repetitive” or “Transcranial Magnetic Stimulation, Single Pulse”or “Transcranial Direct Current Stimulation” or “tDCS” or “Anodal Stimulation Transcranial Direct Current Stimulation” or “Cathodal Stimulation Transcranial Direct Current Stimulation” or “Electric Stimulation” or “Neuromuscular Electrical Stimulation” or “sensory neuromuscular electrical Stimulation” or “Surface Neuro Muscular Electrical Stimulation” or “Therapeutic Electrical Stimulation” or “Therapeutic Electric Stimulation” or “Electrotherapy” or “NMES” or “sNMES” or “acupuncture” or “Electroacupuncture” or “Catheter balloon dilatation” or “balloon dilatation” or “Botulinum toxin injection” or “electromyographic biofeedback” or “electromyography biofeedback” or “Surface electromyography biofeedback” or “Tongue pressure resistance feedback” or “tongue pressure feedback” or “Conventional Dysphagia Therapy” or “Conventional swallowing training” or “swallowing training” or “swallowing exercise” or “swallowing therapy” or “Swallowing rehabilitation therapy”) AND TITLE-ABS-KEY(“randomized controlled trial” or “randomized” or “placebo” or “RCT”) |
| --- |

**Supplementary Table 6.** the search strategy of PROQUEST.

| AB,TI(“stroke” or “strokes” or “Cerebrovascular Accident” or “Cerebrovascular Accidents” or “Cerebral Stroke” or “Cerebral Strokes” or “Stroke, Cerebral” or “Strokes, Cerebral” or “Cerebrovascular Apoplexy” or “Apoplexy, Cerebrovascular” or “Vascular Accident, Brain” or “Brain Vascular Accident” or “Brain Vascular Accidents” or “Vascular Accidents, Brain” or “Cerebrovascular Stroke” or “Cerebrovascular Strokes” or “Stroke, Cerebrovascular” or “Strokes, Cerebrovascular” or “Apoplexy or CVA (Cerebrovascular Accident)” or “CVAs (Cerebrovascular Accident)” or “Stroke, Acute” or “Acute Stroke” or “Acute Strokes” or “Strokes, Acute” or “Cerebrovascular Accident, Acute” or “Acute Cerebrovascular Accidents” or “Cerebrovascular Accidents, Acute”) AND AB,TI(“cricopharyngeal achalasia” or “cricopharyngeal muscle” or “achalasia” or “cricopharyngeal dysfunction” or “Achalasia of the upper esophageal sphincter”) AND AB,TI(“Transcranial Magnetic Stimulation” or “Magnetic Stimulations, Transcranial” or “Magnetic Stimulation, Transcranial” or “Stimulations, Transcranial Magnetic” or “Stimulation, Transcranial Magnetic” or “Transcranial Magnetic Stimulations” or “Transcranial Magnetic Stimulation, Paired Pulse” or “Transcranial Magnetic Stimulation, Repetitive” or “Transcranial Magnetic Stimulation, Single Pulse” or “Transcranial Direct Current Stimulation” or “tDCS” or “Anodal Stimulation Transcranial Direct Current Stimulation” or “Cathodal Stimulation Transcranial Direct Current Stimulation”or “Electric Stimulation” or “Neuromuscular Electrical Stimulation” or “sensory neuromuscular electrical Stimulation” or “Surface Neuro Muscular Electrical Stimulation” or “Therapeutic Electrical Stimulation” or “Therapeutic Electric Stimulation” or “Electrotherapy” or “NMES” or “sNMES” or “acupuncture” or “Electroacupuncture” or “Catheter balloon dilatation” or “balloon dilatation” or “Botulinum toxin injection” or “electromyographic biofeedback” or “electromyography biofeedback” or “Surface electromyography biofeedback” or “Tongue pressure resistance feedback” or “tongue pressure feedback” or “Conventional Dysphagia Therapy” or “Conventional swallowing training” or “swallowing training” or “swallowing exercise” or “swallowing therapy” or “Swallowing rehabilitation therapy”) AND AB,TI(“randomized controlled trial” or “randomized” or “placebo” or “RCT”) |
| --- |

**Supplementary Table 7.** the search strategy of CNKI.

| (SU = '卒中' OR SU = '中风' OR SU = '脑血管意外' OR SU = '脑梗' OR SU = '脑出血' OR SU = '脑缺血') AND (SU = '环咽肌失迟缓' OR SU = '环咽肌失弛缓' OR SU = '食管上括约肌失弛缓' OR SU = '环咽肌功能障碍' OR SU = '环咽肌' OR SU = '失迟缓症') AND (SU = '导尿管球囊扩张术' OR SU = '球囊扩张术' OR SU = '肉毒毒素注射' OR SU = '针灸疗法' OR SU = '针灸' OR SU = '针刺' OR SU = '电针' OR SU = '针法' OR SU = '电刺激' OR SU = '吞咽障碍治疗仪' OR SU = '神经肌肉电刺激' OR SU = '低频脉冲电刺激' OR SU = '神经肌肉低频电刺激' OR SU = '经颅磁刺激' OR SU = '重复经颅磁刺激' OR SU = 'TMS' OR SU = 'rTMS' OR SU = '经颅直流电刺激' OR SU = 'tDCS' OR SU = '肌电生物反馈' OR SU = '肌电生物反馈治疗' OR SU = '肌电生物反馈疗法' OR SU = '表面肌电生物反馈' OR SU = '舌压抗阻反馈训练' OR SU = '舌压反馈训练' OR SU = '常规吞咽治疗' OR SU = '吞咽康复训练' ) AND (AB = '随机对照试验' OR AB = '随机对照实验' OR AB = '随机对照研究' OR AB = 'RCT' OR AB = '随机对照' OR AB = '随机') |
| --- |

**Supplementary Table 8.** the search strategy of Wanfang Database.

| 主题：(卒中 or 中风 or 脑血管意外 or 脑梗 or 脑出血 or 脑缺血) and 主题：(环咽肌失迟缓 or 环咽肌失弛缓 or 环咽肌功能障碍 or 食管上括约肌功能障碍 or 环咽肌 or 失迟缓症) and 主题：(导尿管球囊扩张术 or 球囊扩张术 or 肉毒毒素注射 or 针灸疗法 or 针灸 or 针刺 or 针法 or 电针 or 电刺激 or 吞咽障碍治疗仪 or 神经肌肉电刺激 or 低频脉冲电刺激 or 神经肌肉低频电刺激 or 经颅磁刺激 or 重复经颅磁刺激 or TMS or rTMS or 经颅直流电刺激 or tDCS or 肌电生物反馈 or 肌电生物反馈治疗 or 肌电生物反馈疗法 or 表面肌电生物反馈 or 舌压抗阻反馈训练 or 舌压反馈训练 or 常规吞咽治疗 or 吞咽康复训练) and 主题：(随机对照试验 or 随机对照实验 or 随机对照研究 or RCT or 随机对照 or 随机) |
| --- |

**Supplementary Table 9.** the search strategy of CBM.

| [(("导尿管球囊扩张术"[常用字段:智能] OR "球囊扩张术"[常用字段:智能] OR "肉毒毒素注射"[常用字段:智能]) OR ( "肌电生物反馈"[常用字段:智能] OR "肌电生物反馈治疗"[常用字段:智能] OR "肌电生物反馈疗法"[常用字段:智能] OR "表面肌电生物反馈"[常用字段:智能] OR "舌压抗阻反馈训练"[常用字段:智能] OR "舌压反馈训练"[常用字段:智能] OR "常规吞咽治疗"[常用字段:智能] OR "吞咽康复训练"[常用字段:智能]) OR ( ("tDCS"[常用字段:智能]) OR ("经颅直流电刺激"[不加权:扩展])) OR ( ("重复经颅磁刺激"[常用字段:智能] OR "TMS"[常用字段:智能] OR "rTMS"[常用字段:智能]) OR ("经颅磁刺激"[不加权:扩展])) OR ( ("吞咽障碍治疗仪"[常用字段:智能] OR "神经肌肉电刺激"[常用字段:智能] OR "低频脉冲电刺激"[常用字段:智能] OR "神经肌肉低频电刺激"[常用字段:智能]) OR ("电刺激"[不加权:扩展])) OR ( ("针灸"[常用字段:智能] OR "针刺"[常用字段:智能] OR "针法"[常用字段:智能] OR "电针"[常用字段:智能]) OR ("针灸疗法"[不加权:扩展]))) AND ("环咽肌失迟缓"[常用字段:智能] OR "环咽肌失弛缓"[常用字段:智能] OR "食管上括约肌失弛缓"[常用字段:智能] OR "环咽肌功能障碍"[常用字段:智能] OR "环咽肌"[常用字段:智能] OR "失迟缓症"[常用字段:智能]) AND (("中风"[常用字段:智能] OR "脑血管意外"[常用字段:智能] OR "脑梗"[常用字段:智能] OR "脑出血"[常用字段:智能] OR "脑缺血"[常用字段:智能]) OR ("卒中"[不加权:扩展])) AND (("随机对照实验"[常用字段:智能] OR "随机"[常用字段:智能] OR "随机对照"[常用字段:智能] OR "随机对照研究"[常用字段:智能] OR "RCT"[常用字段:智能]) OR ("随机对照试验"[不加权:扩展]))](javascript:toDoRelimitSearch();) |
| --- |

**Supplementary Table 10.** the search strategy of VIP.

| 题名或关键词 | 卒中+中风+脑血管意外+脑梗+脑出血+脑缺血 |
| --- | --- |
| 题名或关键词 | 环咽肌失迟缓+环咽肌失弛缓+食管上括约肌失弛缓+环咽肌功能障碍+环咽肌+失迟缓症 |
| 题名或关键词 | 导尿管球囊扩张术+球囊扩张术+肉毒毒素注射+针灸疗法+针灸+针刺+针法+电针+电刺激+吞咽障碍治疗仪+神经肌肉电刺激+低频脉冲电刺激+神经肌肉低频电刺激+经颅磁刺激+重复经颅磁刺激+TMS+rTMS+经颅直流电刺激+tDCS+肌电生物反馈+肌电生物反馈治疗+肌电生物反馈疗法+表面肌电生物反馈+舌压抗阻反馈训练+舌压反馈训练+常规吞咽治疗+吞咽康复训练 |
| 摘要 | 随机对照试验+随机对照实验+随机+随机对照+随机对照研究+RCT |

**Supplementary Table 11.** Efficacy evaluation criteria for VFSS.

| VFSS | Cure | The transit times of the oral and pharyngeal cavities are normal. No residual contrast agent is detected in the piriform recesses and valleculae, and there is no aspiration. | Effective rate = (Number of cured cases + Number of effective cases) / Total number of cases × 100% |
| --- | --- | --- | --- |
|  | Effectiveness | The passage time of the oral cavity and pharynx was shorter than that before treatment, and it reached more than 50%. The residual contrast agent in the pyriform sinus and epiglottic fossa decreased. |  |
|  | Ineffectiveness | The passage time of the oral cavity and pharynx was shorter than that before treatment, and it was less than 50%, or the cricopharyngeal muscle did not open as shown by VFSS, and there were errors in swallowing liquid and paste foods. |  |

**Supplementary Table 12.** Efficacy evaluation criteria for the Kubota Water Swallowing Test.

| Kubota drinking water test | Cure | Normal drinking without choking cough, Kubota Water Swallowing Test reaches grade I. | Effective rate = (Number of cured cases + Number of markedly effective cases + Number of effective cases) / Total number of cases × 100% |
| --- | --- | --- | --- |
|  | Marked effectiveness | Kubota Water Swallowing Test reaches grade II, or the treatment improves by 2 grades. |  |
|  | Effectiveness | Kubota Water Swallowing Test reaches grade III, or the improvement after treatment is 2 grades. |  |
|  | Ineffectiveness | There is no significant change in the condition after treatment. |  |

**Supplementary Table 13.** Original studies reporting effective rate outcomes.

| Lin ＆ Zheng (2018) | Using the curative effect evaluation criteria of VFSS |
| --- | --- |
| Huang et al. (2017) |  |
| Huang et al. (2016) |  |
| Tang et al. (2017) |  |
| Zhu et al.(2018) |  |
| Fan et al. (2020) |  |
| Zhao et al. (2016) |  |
| Wang ＆ Liu (2023) | Using the curative effect evaluation criteria of Kubota water swallowing test |
| Zhang (2019) |  |
| Zhang et al. (2021) |  |
| Zhang et al. (2015) |  |
| Zhuang et al. (2015) |  |
| Wang et al. (2021) |  |
| Gao (2020) |  |
| Luo et al. (2020) |  |
| Wang et al. (2023a) |  |
| Jing et al. (2025) |  |
| Wang et al. (2023c) |  |

**Supplementary Table 14.** Quality assessment was conducted using the revised Jadad scale.

| Author & year | random sequence | Allocation concealment | blind method | withdrawal | total points | quality evaluation |
| --- | --- | --- | --- | --- | --- | --- |
| Zhang (2019) | 2 | 1 | 0 | 1 | 4 | high |
| Wang ＆ Liu (2023) | 2 | 1 | 0 | 1 | 4 | high |
| Liu et al. (2023) | 2 | 1 | 0 | 1 | 4 | high |
| He et al. (2016) | 2 | 1 | 0 | 1 | 4 | high |
| Zhang et al. (2025) | 2 | 1 | 0 | 1 | 4 | high |
| Lin ＆ Zheng (2018) | 2 | 1 | 0 | 1 | 4 | high |
| Wang et al. (2023a) | 1 | 1 | 0 | 1 | 3 | low |
| Huang et al. (2017) | 2 | 1 | 0 | 1 | 4 | high |
| Huang et al. (2016) | 2 | 1 | 0 | 1 | 4 | high |
| Long et al. (2021) | 2 | 1 | 0 | 1 | 4 | high |
| Cao et al. (2019) | 1 | 1 | 0 | 1 | 3 | low |
| Yang ＆ Chen (2017) | 2 | 1 | 0 | 1 | 4 | high |
| Mai (2019) | 2 | 1 | 0 | 1 | 4 | high |
| Luo et al. (2020) | 2 | 1 | 0 | 1 | 4 | high |
| Gao (2020) | 2 | 1 | 0 | 1 | 4 | high |
| Zhao et al. (2020) | 1 | 1 | 0 | 1 | 3 | low |
| Wang et al. (2020a) | 1 | 1 | 0 | 1 | 3 | low |
| Zhang et al. (2018) | 2 | 1 | 0 | 1 | 4 | high |
| Wang et al. (2024) | 2 | 1 | 0 | 1 | 4 | high |
| Tang et al. (2017) | 2 | 1 | 0 | 1 | 4 | high |
| Li et al. (2019) | 2 | 1 | 0 | 1 | 4 | high |
| Wang et al. (2021) | 2 | 1 | 0 | 1 | 4 | high |
| Zhang (2017) | 2 | 1 | 0 | 1 | 4 | high |
| Zhuang et al. (2015) | 1 | 1 | 0 | 1 | 3 | low |
| Zhu et al.(2018) | 2 | 1 | 0 | 1 | 4 | high |
| Fan et al. (2020) | 1 | 1 | 0 | 1 | 3 | low |
| Zhang et al. (2015) | 1 | 1 | 0 | 1 | 3 | low |
| Zhao et al. (2016) | 1 | 1 | 0 | 1 | 3 | low |
| Wang et al. (2023b) | 2 | 2 | 0 | 1 | 5 | high |
| Shao et al. (2017) | 2 | 1 | 0 | 1 | 4 | high |
| Wang et al. (2023c) | 2 | 1 | 0 | 1 | 4 | high |
| Wang et al. (2020b) | 2 | 1 | 2 | 1 | 6 | high |
| Xu (2025) | 2 | 1 | 0 | 1 | 4 | high |
| Wei et al. (2016) | 1 | 1 | 0 | 1 | 3 | low |
| Zhang et al. (2021) | 1 | 1 | 0 | 1 | 3 | low |
| Jing et al. (2025) | 2 | 1 | 0 | 1 | 4 | high |

**Supplementary Table 15.** Adverse reaction.

| Study | Sample size （T/C） | adverse reaction | |
| --- | --- | --- | --- |
|  |  | T | C |
| Mai (2019) | 40/40 | One case presented with discomfort and pain in the throat, and another case exhibited mucosal edema in the nasopharynx. | There are no adverse reactions. |
| Zhu et al.(2018) | 25/25 | Two patients presented with water and electrolyte disorders. | Electrolyte imbalance occurred in three patients, and malnutrition occurred in one patient. |
| Wang et al. (2023b) | 25/24 | One patient experienced mild headache at the beginning of repetitive transcranial magnetic stimulation (rTMS) treatment. The symptoms subsided after adaptation, and no further discomfort was reported subsequently. | One case of treatment suspension due to fever. |

# Supplementary Figures

**Supplementary Figure 1**

**
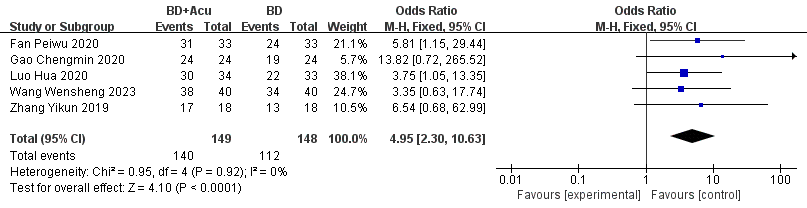
**


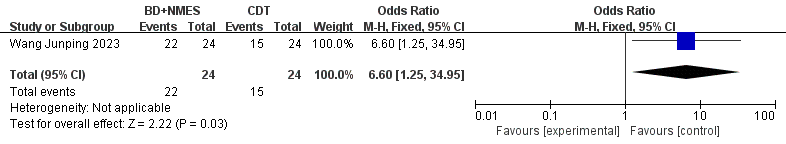


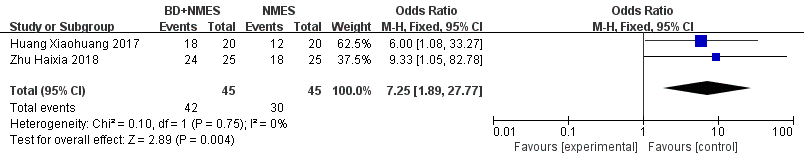


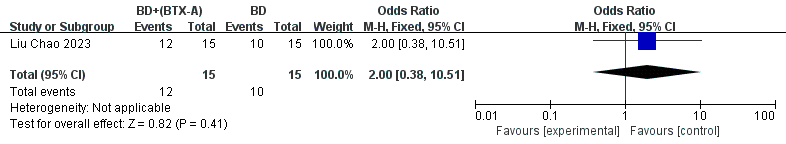


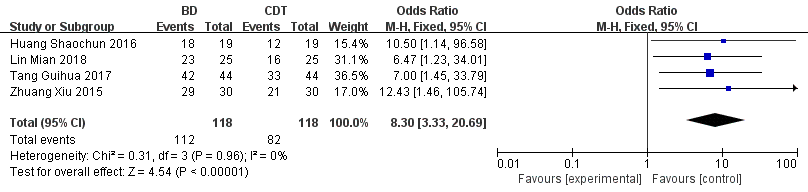


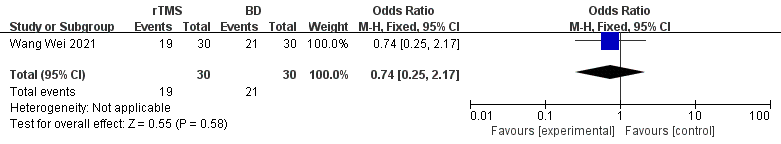


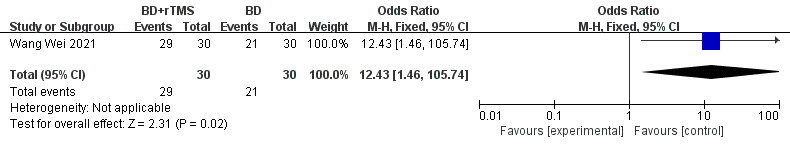


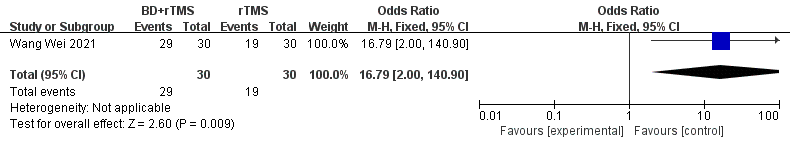


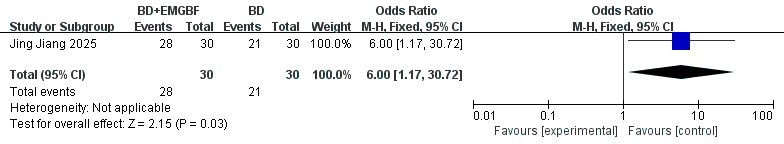

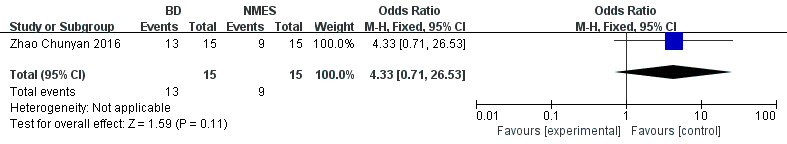


**Supplementary Figure 1.** Forest plot of pairwise Meta-analysis based on effective rate outcome indicators.

**Supplementary Figure 2**


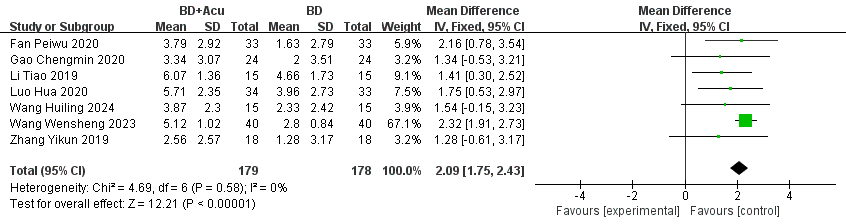


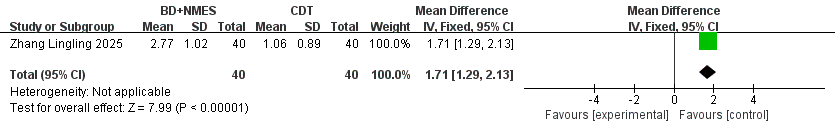


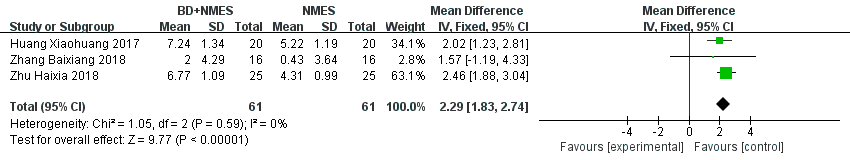


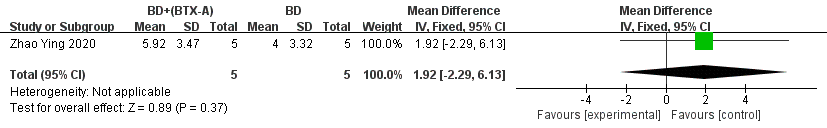


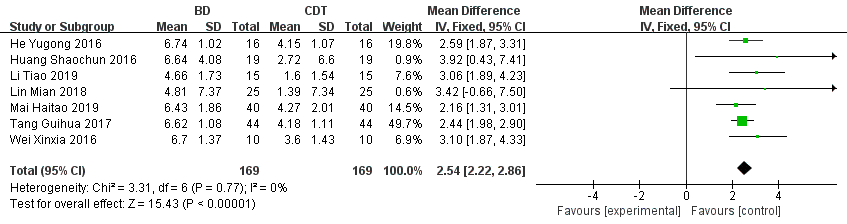


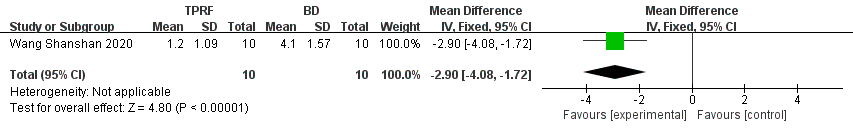


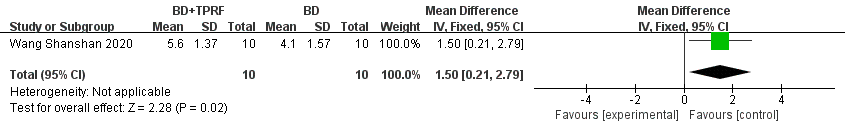


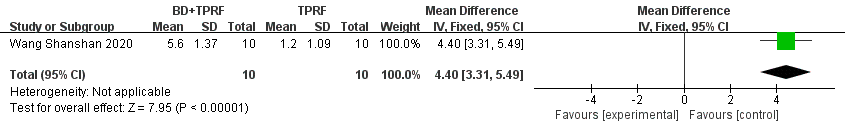


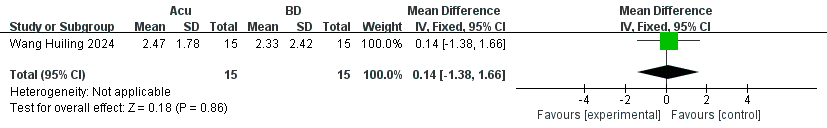


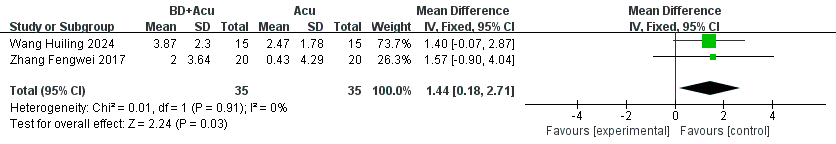


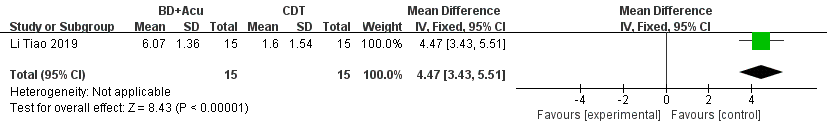


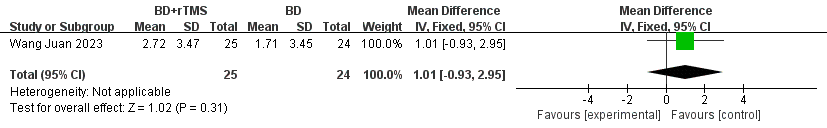


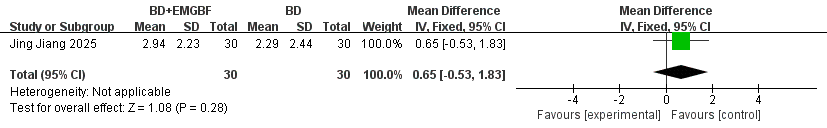


**Supplementary Figure 2.** Forest plot of pairwise Meta-analysis based on Videofluoroscopic Swallowing Study outcome indicators.

**Supplementary Figure 3**


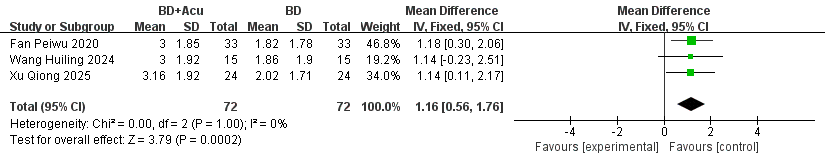


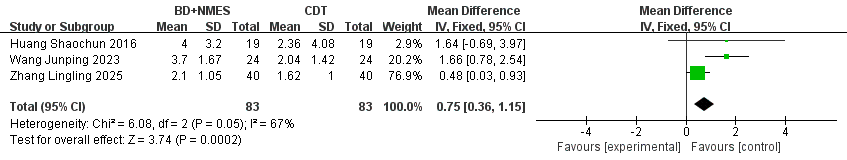


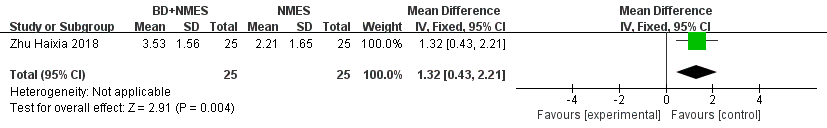


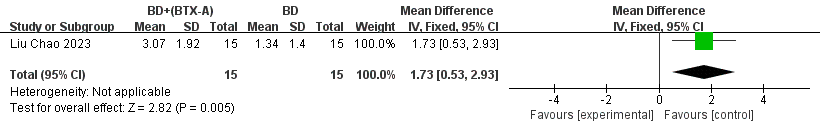


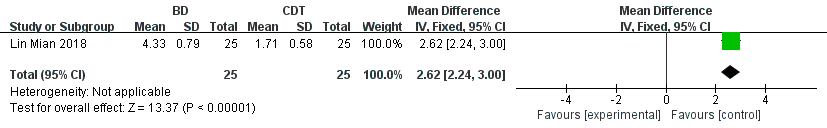


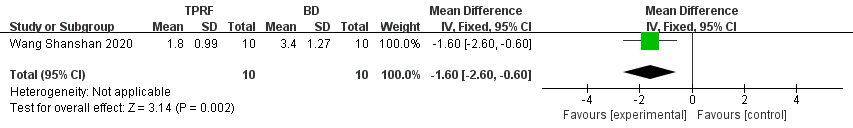


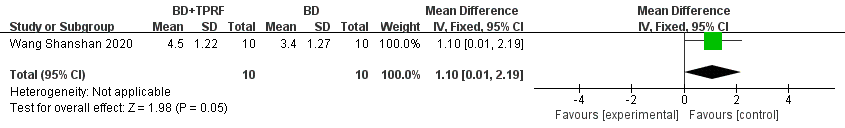


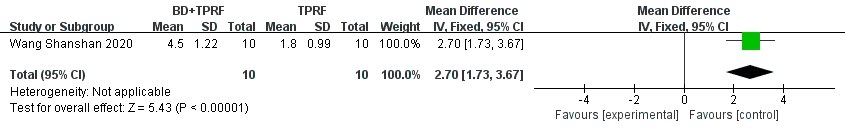


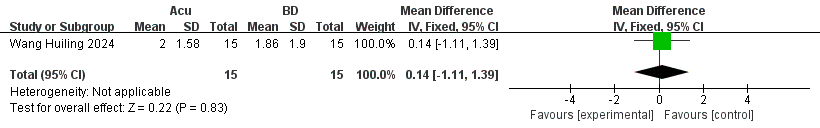


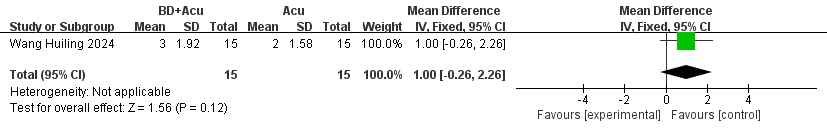


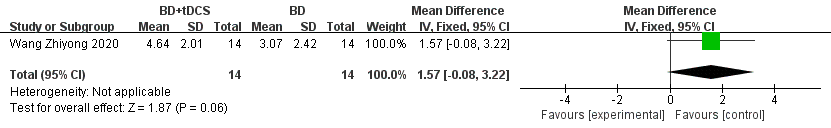


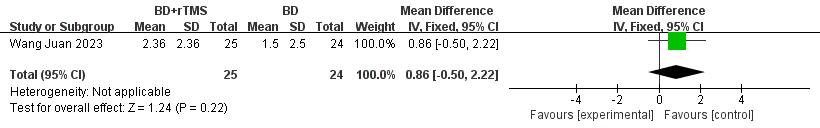


**Supplementary Figure 3.** Forest plot of pairwise Meta-analysis based on Functional Oral Intake Scale outcome indicators.

**Supplementary Figure 4**


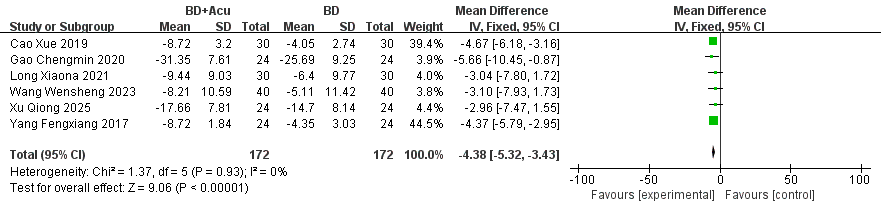


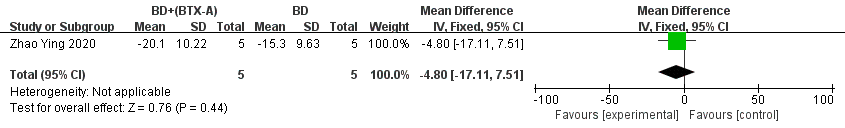


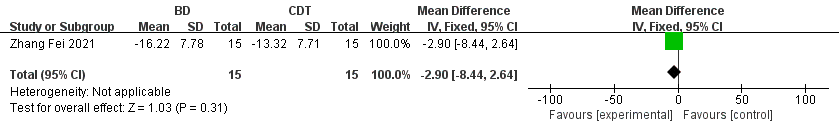


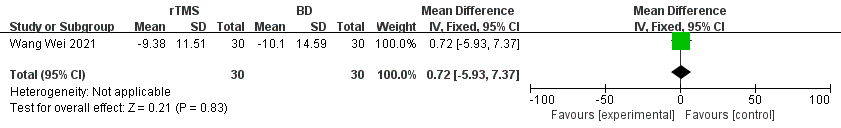


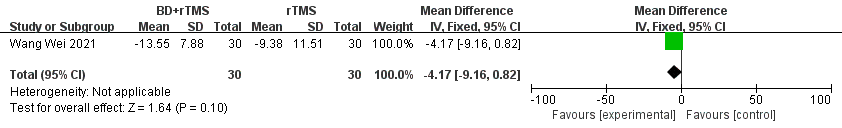


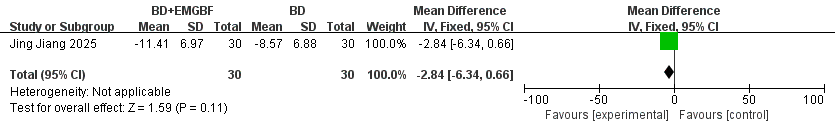


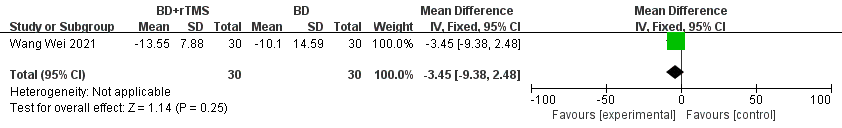


**Supplementary Figure 4.** Forest plot of pairwise Meta-analysis based on Standardized Swallowing Assessment outcome indicators.

**Supplementary Figure 5**


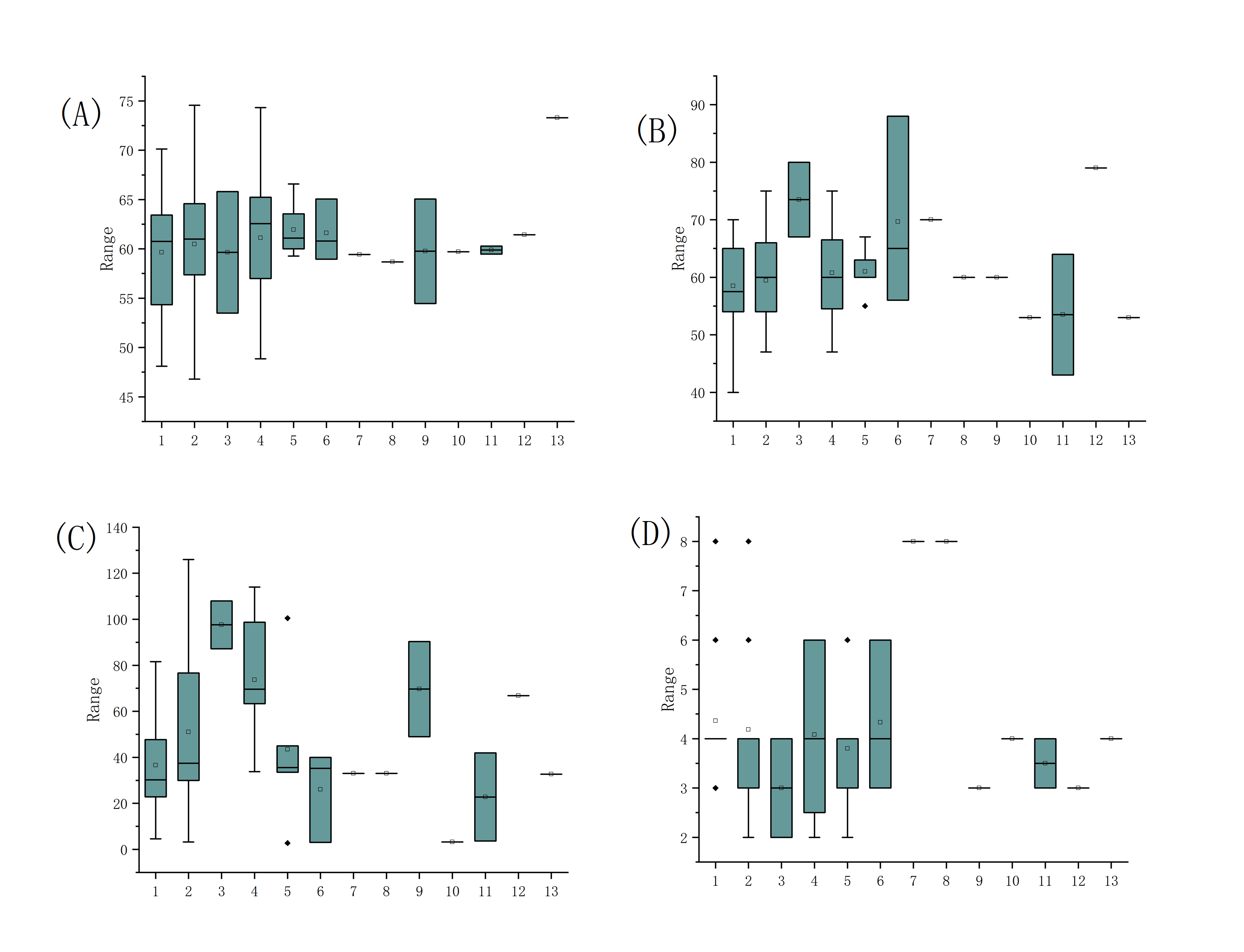


**Supplementary Figure 5.** Box plot for transitivity assessment. (A) represents the mean age, (B) represents the gender ratio, (C) represents the stroke duration, and (D) represents the intervention duration. 1 represents balloon dilation with acupuncture, 2 represents balloon dilation, 3 represents balloon dilation with botulinum toxin type A, 4 represents conventional dysphagia training, 5 represents balloon dilation with neuromuscular electrical stimulation, 6 represents neuromuscular electrical stimulation, 7 represents tongue pressure resistance feedback, 8 represents balloon dilation with tongue pressure resistance feedback, 9 represents acupuncture, 10 represents repetitive transcranial magnetic stimulation, 11 represents balloon dilation with repetitive transcranial magnetic stimulation, 12 represents balloon dilation with transcranial direct current stimulation, 13 represents balloon dilation with electromyographic biofeedback.

**Supplementary Figure 6**

**a**


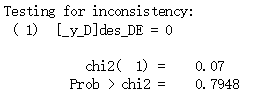


**b**


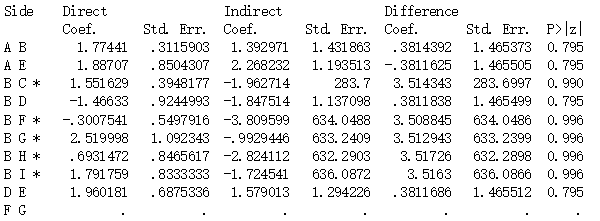


**c**


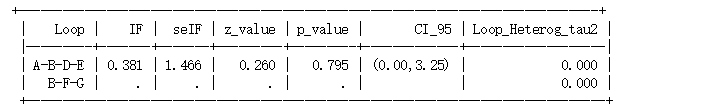


**Supplementary Figure 6.** Inconsistency test based on effective rate outcome indicators. (**a**) overall inconsistency test; (**b**) Local inconsistency tests; (**c**) loop inconsistency test; In the figure, A represents conventional dysphagia training, B represents balloon dilation, C represents balloon dilation combined with acupuncture, D represents neuromuscular electrical stimulation, E represents balloon dilation combined with neuromuscular electrical stimulation, F represents repetitive transcranial magnetic stimulation, G represents balloon dilation combined with repetitive transcranial magnetic stimulation, H represents balloon dilation combined with botulinum toxin type A, I represents balloon dilation combined with electromyographic biofeedback.

**Supplementary Figure 7**

**a**


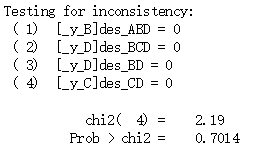


**b**


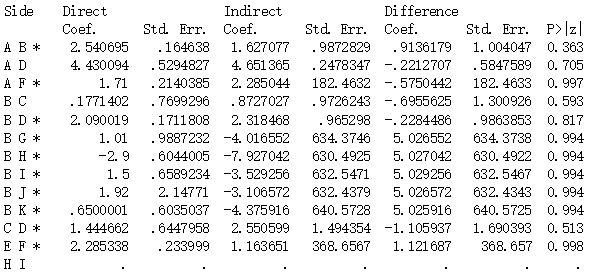


**c**


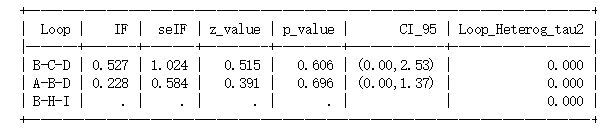


**Supplementary Figure 7.** Inconsistency test based on videofluoroscopic swallowing study outcome indicators. (**a**) overall inconsistency test; (**b**) Local inconsistency tests; (**c**) loop inconsistency test; In the figure, A represents conventional dysphagia training, B represents balloon dilation, C represents acupuncture, D represents balloon dilation combined with acupuncture, E represents neuromuscular electrical stimulation, F represents balloon dilation combined with neuromuscular electrical stimulation, G represents balloon dilation combined with repetitive transcranial magnetic stimulation, H represents tongue pressure resistance feedback, I represents balloon dilation combined with tongue pressure resistance feedback, J represents balloon dilation combined with botulinum toxin type A, K represents balloon dilation combined with electromyographic biofeedback.

**Supplementary Figure 8**

**a**


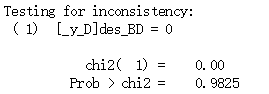


**b**


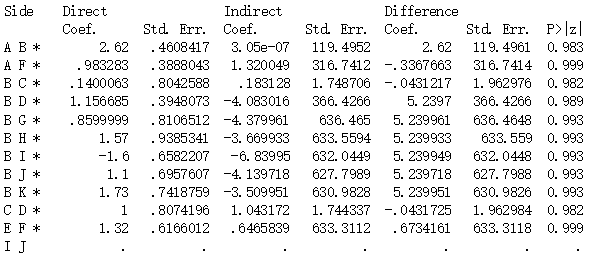


**c**


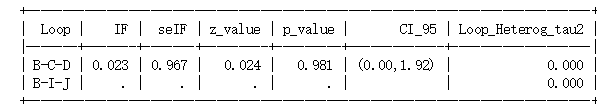


**Supplementary Figure 8.** Inconsistency test based on functional oral intake scale outcome indicators. (**a**) overall inconsistency test; (**b**) Local inconsistency tests; (**c**) loop inconsistency test; In the figure, A represents conventional dysphagia training, B represents balloon dilation, C represents acupuncture, D represents balloon dilation combined with acupuncture, E represents neuromuscular electrical stimulation, F represents balloon dilation combined with neuromuscular electrical stimulation, G represents balloon dilation combined with repetitive transcranial magnetic stimulation, H represents balloon dilation combined with transcranial direct current stimulation, I represents tongue pressure resistance feedback, J represents balloon dilation combined with tongue pressure resistance feedback, K represents balloon dilation combined with botulinum toxin type A.

**Supplementary Figure 9**


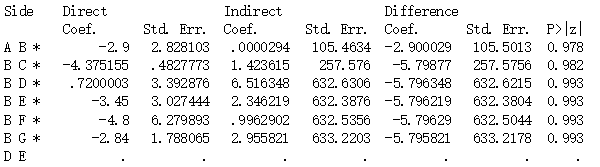


**Supplementary Figure 9.** Inconsistency test based on standard swallowing assessment outcome indicators. Local inconsistency tests; In the figure, A represents conventional dysphagia training, B represents balloon dilation, C represents balloon dilation combined with acupuncture, D represents repetitive transcranial magnetic stimulation, E represents balloon dilation combined with repetitive transcranial magnetic stimulation, F represents balloon dilation combined with botulinum toxin type A, G represents balloon dilation combined with electromyographic biofeedback.

**Supplementary Figure 10**


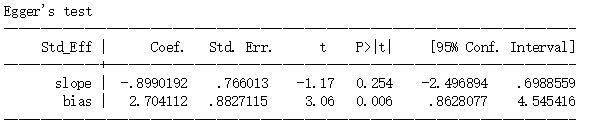


**Supplementary Figure 10.** Results of Egger's test with effective rate scores.

**Supplementary Figure 11**


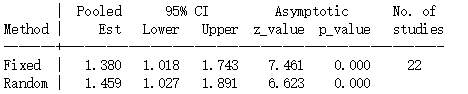


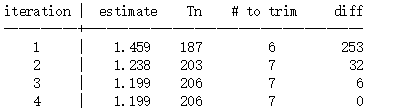


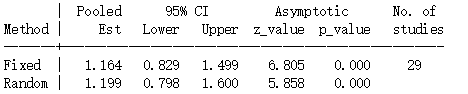


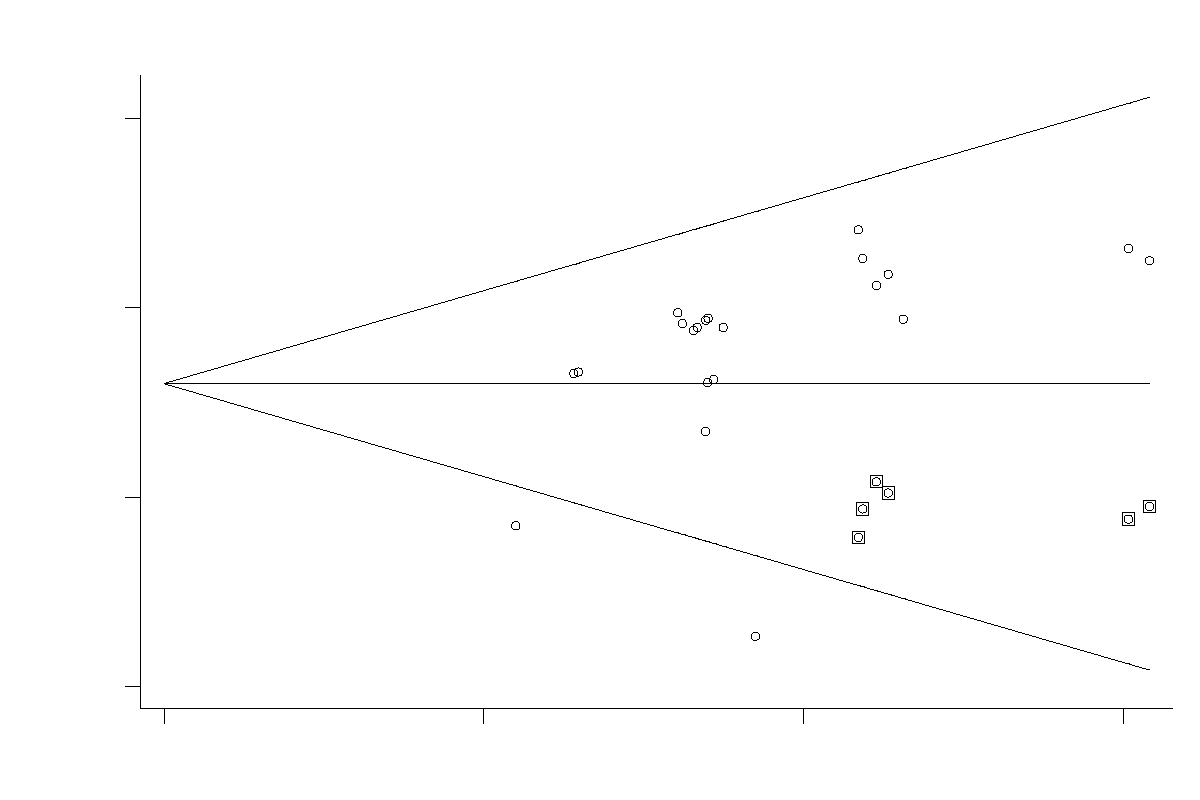


**Supplementary Figure 11.** effective rate score pruning and supplementation corrected funnel plot. For the funnel plot of the effective rate of the outcome indicator, the trim-and-fill method was employed for correction. After four iterations, a total of seven virtual studies were generated. Prior to the trim-and-fill procedure, 22 studies were included, with an effect size of 1.459 (95% CI: 1.027 - 1.891). After the trim-and-fill adjustment, the total number of studies increased to 29, and the effect size was 1.199 (95% CI: 0.798 - 1.600). The results suggest the possible presence of mild publication bias.

**Supplementary Figure 12**


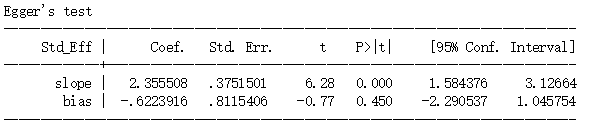


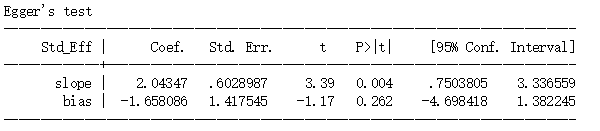


**Supplementary Figure 12.** Results of Egger's test for VFSS scores and FOIS scores.

**Supplementary Figure 13**


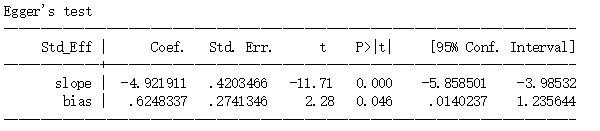


**Supplementary Figure 13.** Results of Egger's test for SSA scores.

**Supplementary Figure 14**


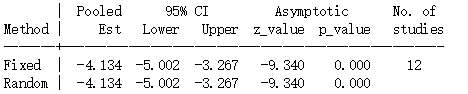


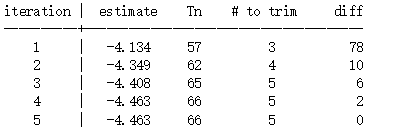


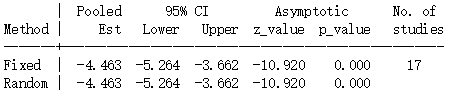


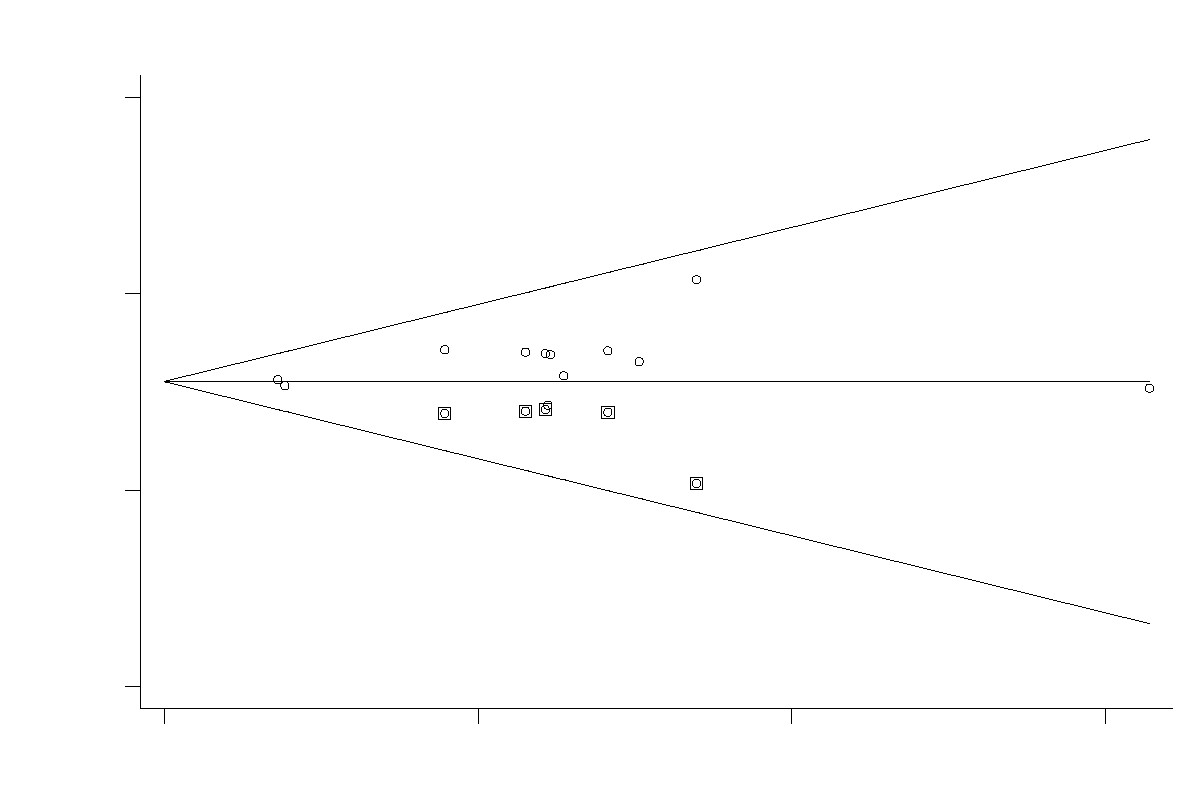


**Supplementary Figure 14.** Funnel plot with SSA score pruning and supplementation correction. For the funnel plot of the outcome indicator SSA, the trim-and-fill method was employed for correction. After five iterative calculations, a total of five virtual research literatures were ultimately generated. Before the trim-and-fill procedure, 12 literatures were included, and the pooled effect size was -4.134 (95% CI: -5.002 to -3.267). After the trim-and-fill procedure, the total number of literatures increased to 17, and the pooled effect size was -4.463 (95% CI: -5.264 to -3.662). The results indicate a possibility of mild publication bias.
